# Local Growth Hormone Facilitates Aging of the Colon Epithelial Microenvironment

**DOI:** 10.1111/acel.70187

**Published:** 2025-08-05

**Authors:** Vera Chesnokova, Svetlana Zonis, Richard Ainsworth, Tugce Apaydin, Christian Wong Valencia, Elora C. Greiner, Robert Barrett, Arminja N. Kettenbach, Shlomo Melmed

**Affiliations:** ^1^ Department of Medicine, Division of Endocrinology, Metabolism and Diabetes Cedars‐Sinai Medical Center Los Angeles California USA; ^2^ Department of Medicine, Kao Autoimmunity Institute Cedars‐Sinai Medical Center Los Angeles California USA; ^3^ Board of Governors Regenerative Medicine Institute Cedars‐Sinai Medical Center Los Angeles California USA; ^4^ Department of Biochemistry and Cell Biology Geisel School of Medicine at Dartmouth Hanover New Hampshire USA; ^5^ Dartmouth Cancer Center Lebanon New Hampshire USA

**Keywords:** aging, epithelial‐mesenchymal transition, extracellular matrix, growth hormone

## Abstract

Aging is associated with the appearance of senescent cells secreting the senescence‐associated secretome, facilitating a milieu favoring age‐related microenvironmental changes. As we previously showed the production of local nonpituitary growth hormone (npGH) in senescent colon epithelial cells, we now elucidate mechanisms underlying npGH action in the nontumorous colon tissue microenvironment. We demonstrate autocrine npGH action in normal human colon cells (hNCC) infected with lentivirus‐expressing hGH (lentiGH), as well as paracrine npGH action in hNCC cocultured with lentiGH hNCC and in intact human 3‐dimensional intestinal organoids cocultured with organoids infected with lentiGH. Enriched gene ontology and pathway analysis of intact organoids exposed to paracrine npGH identified distorted extracellular matrix (ECM) and focal adhesion pathways concurrent with altered expression of ECM and cytoskeletal proteins. Significant phosphoprotein changes associated with the cytoskeleton and cell migration pathway occurred in GH‐exposed hNCC. Paracrine npGH triggers these changes by activating epithelial‐mesenchymal transition, as shown by suppression of E‐cadherin and induction of Twist2 in cellular models, as well as in the colon of nude mice inoculated with GH‐secreting xenografts. These changes are consistent with observed increased migration of hNCC overexpressing lentiGH, or in those cocultured with GH‐secreting hNCC or with GH‐secreting normal colon fibroblasts. Furthermore, whole exome sequencing detected increased structural variation in intact organoids cocultured with lentiGH‐infected organoids, likely as a consequence of GH‐mediated suppressed DNA damage repair, thereby favoring cell transformation. Our results indicate that local growth hormone facilitates aging of the colon epithelial microenvironment.

## Graphical abstract

Local growth hormone (GH) is expressed and secreted by senescent cells in the aging human colon. GH triggers epithelial‐to‐mesenchymal transition and activates colon cell motility by regulating extracellular matrix proteins, inducing cytoskeleton rearrangement and altering cell motility protein phosphorylation. By suppressing DNA repair, GH enables chromosomal instability with subsequent increased mutations, favoring cell transformation. Thus, local GH facilitates aging of the colon epithelial microenvironment.

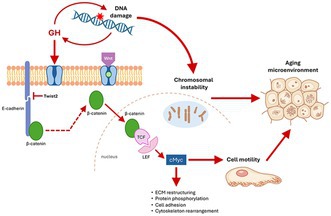

## 1 Introduction

The tissue microenvironment comprises immune‐, stem‐, endothelial, stromal‐, and epithelial cells, and extracellular matrix (ECM). Altered cellular composition or intercellular communication may markedly affect microenvironmental tissue homeostasis. Such microenvironmental changes affect tumor growth and progression (Campbell and Booth 2023; de Visser and Joyce 2023; Fane and Weeraratna 2020), but there is limited knowledge about the normal tissue microenvironment and mechanisms creating a milieu favorable for subsequent age‐associated neoplastic development. Age‐associated changes include genetic and epigenetic alterations, inflammation (Chen et al. 2021; Ryu et al. 2016), and ECM‐dependent tissue architecture (Almagro et al. 2022), as well as somatic mutations occurring as a result of unrepaired DNA damage and chromosomal instability (Lopez‐Otin et al. 2023).

Epithelial–mesenchymal transition (EMT) likely plays an initial role allowing polarized epithelial cells, normally attached to the basement membrane, to undergo changes consistent with a mesenchymal cell phenotype. These cells show enhanced migratory capacity and apoptosis resistance, as well as increased production of ECM components (Kalluri and Neilson 2003; Kalluri and Zeisberg 2006). EMT is sensitive to and activated by DNA damage (Moyret‐Lalle et al. 2022). With age, DNA repair is weakened (Feng et al. 2007; Gutierrez‐Martinez et al. 2018), and unrepaired damage or faulty repair may facilitate acquisition of prooncogenic mutations, as well as accumulation of senescent cells. Senescence is initially protective, restraining uncontrolled proliferation of potentially tumorigenic cells (Di Micco et al. 2021; Gorgoulis et al. 2019). However, senescence also regulates the cell microenvironment through secretion of the senescence‐associated secretory phenotype (SASP), comprising matrix metalloproteinases (MMPs) that degrade ECM, as well as growth factors, proinflammatory cytokines, and chemokines (Herbstein et al. 2024) Thus, eliminating senescent cells or blocking SASP may ameliorate adverse age‐related microenvironment changes (Baker et al. 2011; Childs et al. 2015). Senolytic drugs targeting these cells are proposed to treat age‐related disorders (Chaib et al. 2022; Kirkland and Tchkonia 2020).

We recently showed that local, nonpituitary growth hormone (npGH) is expressed in senescent cells and is a SASP component exhibiting autocrine and paracrine actions. By contrast, circulating pituitary‐derived GH activates liver insulin‐like growth factor 1 (IGF1), and the GH/IGF1 axis induces skeletal growth and elicits anabolic and metabolic functions in adults (Chia 2014; Chikani and Ho 2014; Clayton et al. 2014; Faje and Barkan 2010; Giustina et al. 2008; Lanning and Carter‐Su 2006; Yakar and Isaksson 2016). Although circulating pituitary‐derived GH declines markedly with age (Bartke 2008; Chapman et al. 1997; Milman et al. 2016), we observed npGH accumulation within aging human colon tissue and in aged 3‐dimensional induced pluripotent stem cell (iPSC)‐derived human intestinal organoids, as well as in human fibroblasts derived from patients with progeria (Chesnokova et al. 2021). Further, paracrine‐acting npGH, consistent with its SASP function, signals to neighboring cells, triggering proliferation, inactivating tumor‐suppressing p53, and enhancing DNA damage accumulation by suppressing DNA damage repair (Chesnokova et al. 2024). We also found that GH promotes cell transformation and metastases in immunocompromised mice harboring GH‐secreting xenografts and injected intrasplenically with postsenescent human nontumorous colon cells (Chesnokova et al. 2024).

As accumulated SASP‐secreting senescent cells promote preneoplastic cell properties often occurring in aging tissues (Calcinotto et al. 2019; Lee and Schmitt 2019), we hypothesized that autocrine/paracrine npGH activity we observed in nontransformed epithelial cells is a component of the microenvironment facilitating age‐associated changes. We therefore studied autocrine npGH action in normal human colon cells (hNCC) derived from surgical specimens and modeled npGH SASP effects by coculturing hNCC expressing GH with intact hNCC. We found that autocrine and paracrine GH activates EMT, with E‐cadherin suppression and nuclear β‐catenin translocation, resulting in cMyc activation, increased MMP2 and MMP9 expression, and increased cell motility. We confirmed these observations in vivo, showing increased colon Twist2 and suppressed E‐cadherin in nude mice harboring GH‐secreting xenografts.

We further assessed paracrine effects of local GH on neighboring cells by infecting organoids with lentivirus expressing GH (lentiGH). We found that, similar to hNCC, organoids exposed to paracrine GH exhibit activated EMT. RNA‐seq identified regulatory pathways and potential mechanisms for paracrine GH in the normal aging tissue microenvironment. Enriched gene ontology (GO), Reactome, and Kyoto Encyclopedia of Genes and Genomes (KEGG) pathway analyses revealed altered ECM structure and focal adhesion pathways in organoid cells exposed to paracrine GH. Confirmed genomic changes with real‐time (RT)‐PCR and Western blotting showed altered genes and proteins associated with ECM as well as with cell cytoskeletal rearrangements. Moreover, phospho‐omics revealed phosphorylation changes in proteins associated with cytoskeletal rearrangement and migration pathways. Global gene expression changes in intact human intestinal organoids showed that paracrine GH exposure increased structural variations (SV) that may arise as a consequence of DNA damage. Thus, our results show that accumulated and locally secreted npGH facilitate aging of the colon microenvironment.

## 2 Results

### 2.1 Aging Human Colon Tissue npGH Is Expressed in Senescent Cells

**FIGURE 1:**
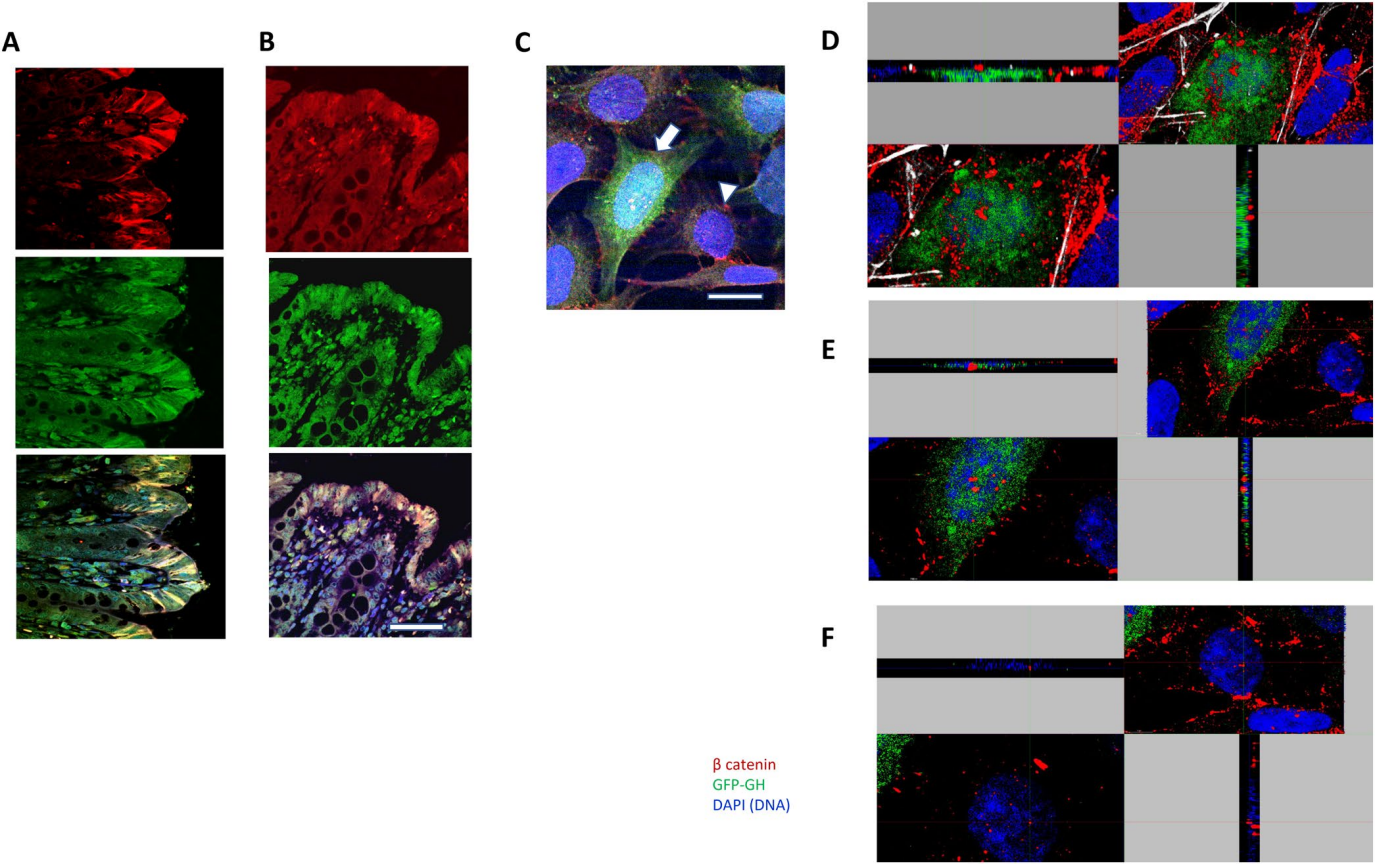
The npGH is expressed in senescent cells, and autocrine and paracrine GH promote β‐catenin nuclear translocation. (A‐B) Representative confocal images of npGH expression in senescent cells in (A) normal colon and (B) hyperplastic colon polyp. SA‐β‐gal, green; GH, red; colocalization, yellow. Scale bar = 100 μm. (C) Representative confocal image of hNCC expressing lentiGH. Arrow indicates GFP‐positive cell expressing GH (green) and active β‐catenin (red). Arrowhead indicates the cell cultured in proximity of a GH‐expressing cell also expressing intranuclear active β‐catenin. Scale bar = 20 μm. (D‐F) Representative cross‐sections of confocal Z‐stacks of hNCC showing localization of active β‐catenin (red) and nucleus (blue). (D) Active β‐catenin is outside the nucleus in GFP‐positive lentiV hNCC, (E) in the nucleus of GFP‐positive lentiGH hNCC, and (F) in the nucleus of GFP‐negative hNCC cocultured with lentiGH hNCC.

We confirmed (Chesnokova et al. 2024, 2021) that npGH expression in human colon tissue increases with age (Figure S1A). We now show that SA‐β‐gal activity in senescent cells correlates with β‐galactosidase expression (Kurz et al. 2000; Lee et al. 2006), and that npGH colocalizes with this senescence marker in resected nontumorous colon tissue (Figure 1A) and in hyperplastic colon polyps (Figure 1B). While GH is not expressed in most colon epithelial cells expressing Ki67, a proliferation marker, it colocalizes with p16 (Figure S1B), confirming that GH is expressed in nonproliferating cells.

### 2.2 Autocrine npGH Triggers hNCC EMT and β‐Catenin Nuclear Translocation

We assessed autocrine actions of npGH on EMT in hNCC infected with GFP‐expressing lentiGH or empty vector (lentiV) and imaged confocally 1 week later. Figure 1C depicts a cell infected with GFP‐lentiGH and non‐infected cells cultured in close proximity with a GH‐secreting cell. hNCC infected with lentiV hNCC did not show intranuclear β‐catenin localization (Figure 1D). By contrast, in lentiGH hNCC, β‐catenin was translocated to the nucleus as evidenced by coexpression with DNA (Figure 1E). Intranuclear β‐catenin was also present in intact GFP‐negative cells growing in close proximity to lentiGH hNCC (Figure 1F), indicative of npGH paracrine action.

**FIGURE 2:**
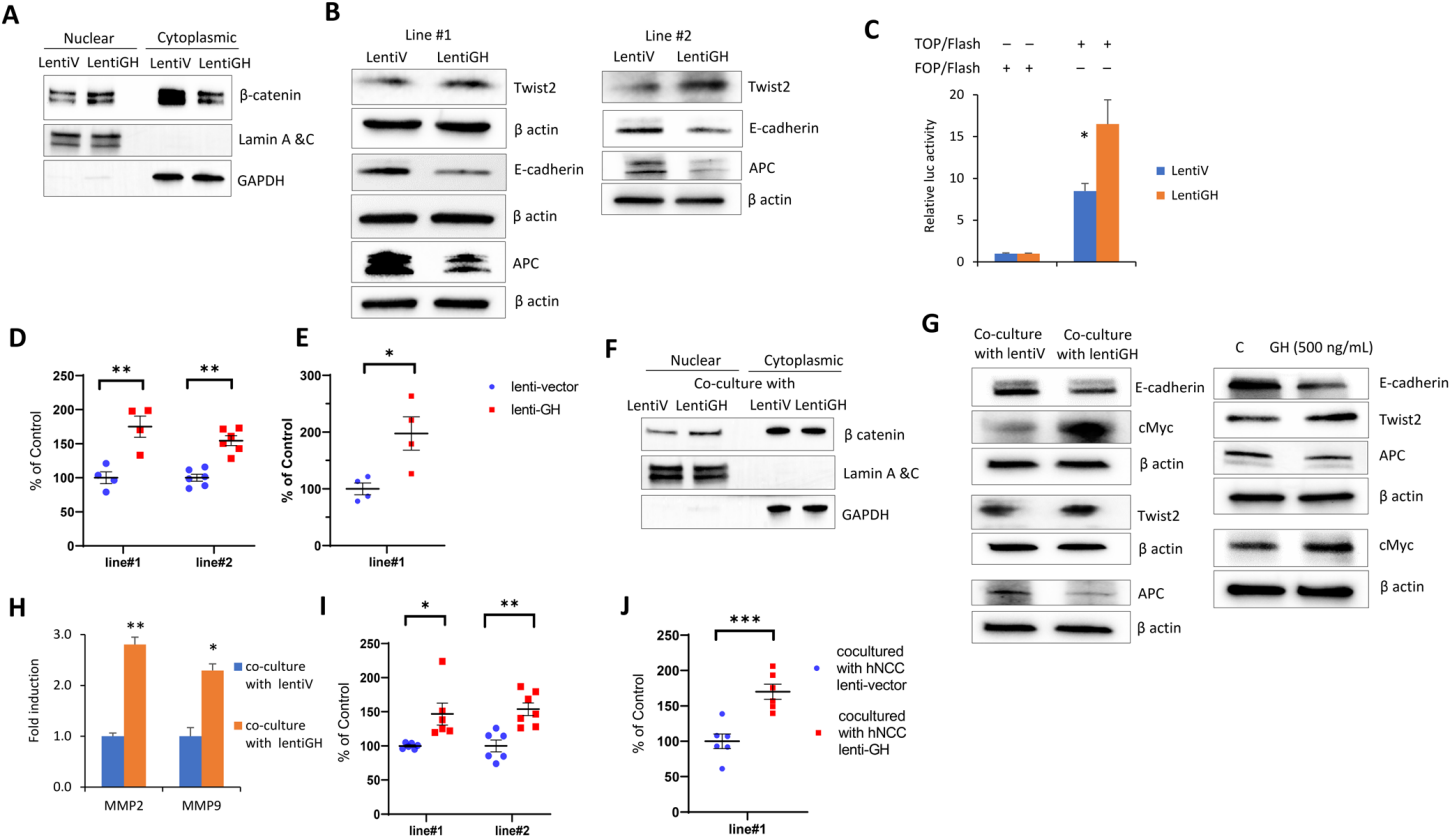
Autocrine and paracrine GH trigger EMT and β‐catenin nuclear translocation. (A‐E) Autocrine GH triggers EMT and β‐catenin nuclear translocation. (A) Cytoplasmic (CF) and nuclear (NF) fractions of hNCC infected with lentiV or lentiGH and analyzed 7 days after infection. ImageJ quantification of protein expression is depicted on Figure S2. (B) EMT markers in hNCC line #1 and line #2 infected with lentiV or lentiGH. (C) Assay of canonical Wnt pathway signaling through activation of a TCF/LEF luciferase reporter construct (TOPFlash) or a control reporter (FOPFlash). hNCC infected with lentiV or lentiGH were nucleofected with reporter constructs. Luciferase activity was normalized to pRL‐TK Renilla Luciferase Control Reporter Vector. Relative luciferase activity was calculated as the ratio between light units of TOPFlash divided by those of FOPFlash. (D) Migration of hNCC line#1 and line #2 infected with lentiV or lentiGH. (E) Invasion of hNCC line #1 infected with lentiV or lentiGH. Graph depicts duplicates of two or three experiments. Microscope images are depicted in Figure S2D,E. (F–J) Paracrine GH triggers EMT and β‐catenin nuclear translocation. (F) Cytoplasmic and nuclear fractions of hNCC cocultured with hNCC infected with lentiV or lentiGH and analyzed 7 days later. (G) EMT markers in hNCC cocultured with hNCC infected with lentiV or lentiGH (left) or treated with 500 ng/mL GH (right) for 24 h. (H) RT‐PCR of MMPs in hNCC cocultured with hNCC infected with lentiV or lentiGH for 1 month. (I) Migration of hNCC line#1 and line#2 cocultured with hNCC infected with lentiV or lentiGH. (J) Invasion of hNCC line#1 cocultured with hNCC infected with lentiV or lentiGH. In I, J graphs present duplicates of three experiments. In D, E, I, J results are depicted as mean ± SEM. *p < 0.05, **p < 0.01. Microscope images of migration and invasion are depicted in Figure S4E,F. ImageJ quantifications of protein expression are depicted in Figure S2A–C and Figure S4A,B.

Confirming these results by Western blotting, we showed increased nuclear active β‐catenin expression, with decreased cytoplasmic expression (Figure 2A and Figure S2A). Consistent with these results, we observed increased Twist2 and suppressed E‐cadherin and APC in these cells and also in an additional hNCC (line#2) derived from a different patient (Figure 2B and Figure S2C). Our results were confirmed by demonstrating modest but consistently increased β‐catenin transcriptional activity in lentiGH hNCC through T‐cell factor/lymphoid enhancer binding factor (TCF/LEF) binding sites (Figure 2C).

EMT activation by GH was also evidenced by markedly increased migration and invasion of lentiGH hNCC versus lentiV hNCC (Figure 2D,E and Figure S2D,E). These results were supported by the appearance of long cellular extensions in lentiGH hNCC indicative of cell migration (Figure S3).

### 2.3 Paracrine hNCC npGH Triggers EMT and β‐Catenin Nuclear Translocation

To assess paracrine npGH effects, intact hNCC were cocultured with lentiGH hNCC or lentiV hNCC for 1 week. In cells exposed to paracrine GH, cytoplasmic β‐catenin decreased while intranuclear β‐catenin increased (Figure 2F and Figure S4A). Paracrine npGH also induced Twist2, resulting in suppressed E‐cadherin and APC (Figure 2G and Figure S4B). Similar changes were seen in cells cocultured with lentiGH hNCC line #2 (Figure S4C,D). After GH exposure, β‐catenin targets, including proto‐oncogene c‐Myc (Figure 2G) and matrix metalloproteinase 2 (MMP2) and MMP9 mRNA, both involved in ECM degradation and cell migration (Figure 2H), were upregulated, concordant with β‐catenin nuclear translocation. Treatment with GH (500 ng/mL) for 24 h resulted in effects consistent with paracrine GH action (Figure 2G). Similar to autocrine npGH effects, paracrine npGH enhanced migration in both hNCC line #1 and line #2 as well as invasion in line#1 (Figure 2I,J and Figure S4E,F).

**FIGURE 3:**
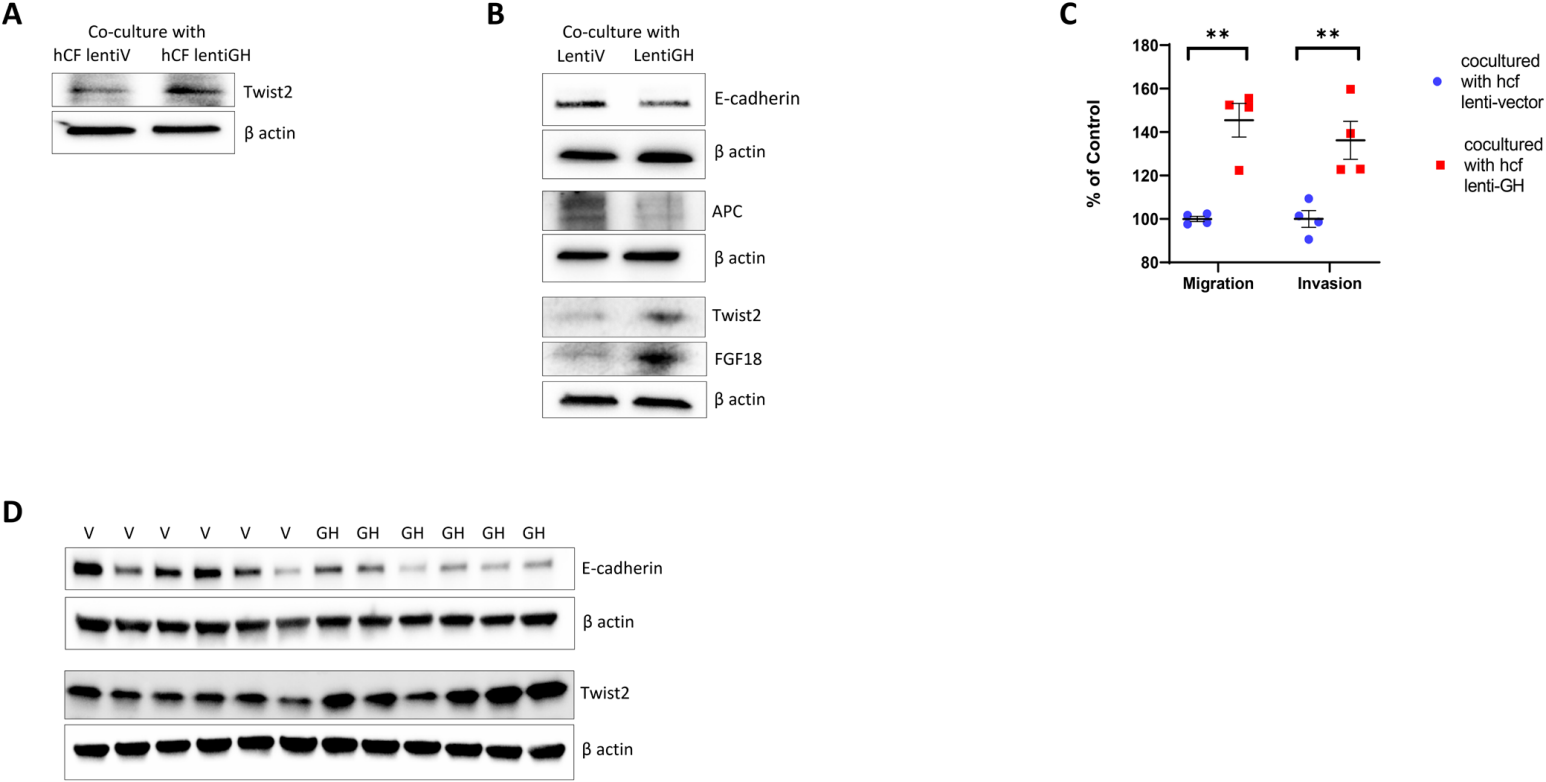
Paracrine GH induces EMT markers in human colon fibroblast, organoids, and murine colon. (A) Twist2 expression in intact hNCC cocultured with hCF infected with lentiV or lentiGH. (B) EMT markers in organoids cocultured for 1 month with organoids expressing lentiV or lentiGH. (C) Migration and invasion of hNCC cocultured with hCF infected with lentiV or lentiGH. Graph presents duplicates of two experiments. Microscope images of migration and invasion are depicted in Figure S5. (D) EMT markers in the colon tissue of mice carrying GH‐secreting or vector‐secreting xenografts. ImageJ quantification of protein expression for B and D is depicted Figure S6.

As npGH is also induced in aging stroma (Figure S1), we examined the effects of paracrine GH derived from normal human colon fibroblasts (hCF) infected with lentiGH. Coculture of intact hNCC with lentiGH hCF for 4 days resulted in Twist2 upregulation (Figure 3A) as well as increased migration and invasion (Figure 3C and Figure S5A,B) as compared to hNCC cocultured with lentiV hCF.

### 2.4 Paracrine npGH Triggers EMT in Human Intestinal Organoids

As GH is a SASP component markedly induced in senescent organoids (Chesnokova et al. 2024, 2021), we tested paracrine GH action in intact organoids cocultured for 1 month together with organoids infected with lentiGH. Paracrine GH suppressed E‐cadherin, decreased APC, and induced Twist2 expression compared to organoids cocultured with those expressing lentiV. Fibroblast growth factor 18 (FGF18), a mesenchymal marker, was also induced in organoids subjected to paracrine GH (Figure 3B and Figure S6A).

### 2.5 Paracrine GH Triggers EMT in Murine Colon Tissue

To further explore in vivo paracrine GH influence on EMT, we inoculated athymic male nude mice subcutaneously with human colon adenocarcinoma HCT116 cells stably expressing mGH or empty vector. We previously showed that HCT116 xenografts form GH‐secreting tumors, achieving ~40‐fold increased circulating GH levels (Chesnokova et al. 2024, 2016). After five weeks, mice were sacrificed and E‐cadherin was observed to be suppressed while Twist2 was induced in colon tissue of mice exposed to high circulating GH (Figure 3D and Figure S6B). By contrast, colon EMT markers were unchanged in GH receptor knockout (GHRKO) mice with disrupted GH signaling (Figure S7A,B).

### 2.6 Paracrine GH Alters ECM, Cytoskeleton, and Focal Adhesion Transcriptional Pathways

To elucidate mechanisms for paracrine GH actions, 3 independent organoid lines were infected with lentiV or lentiGH and cocultured for 1 month.

**FIGURE 4:**
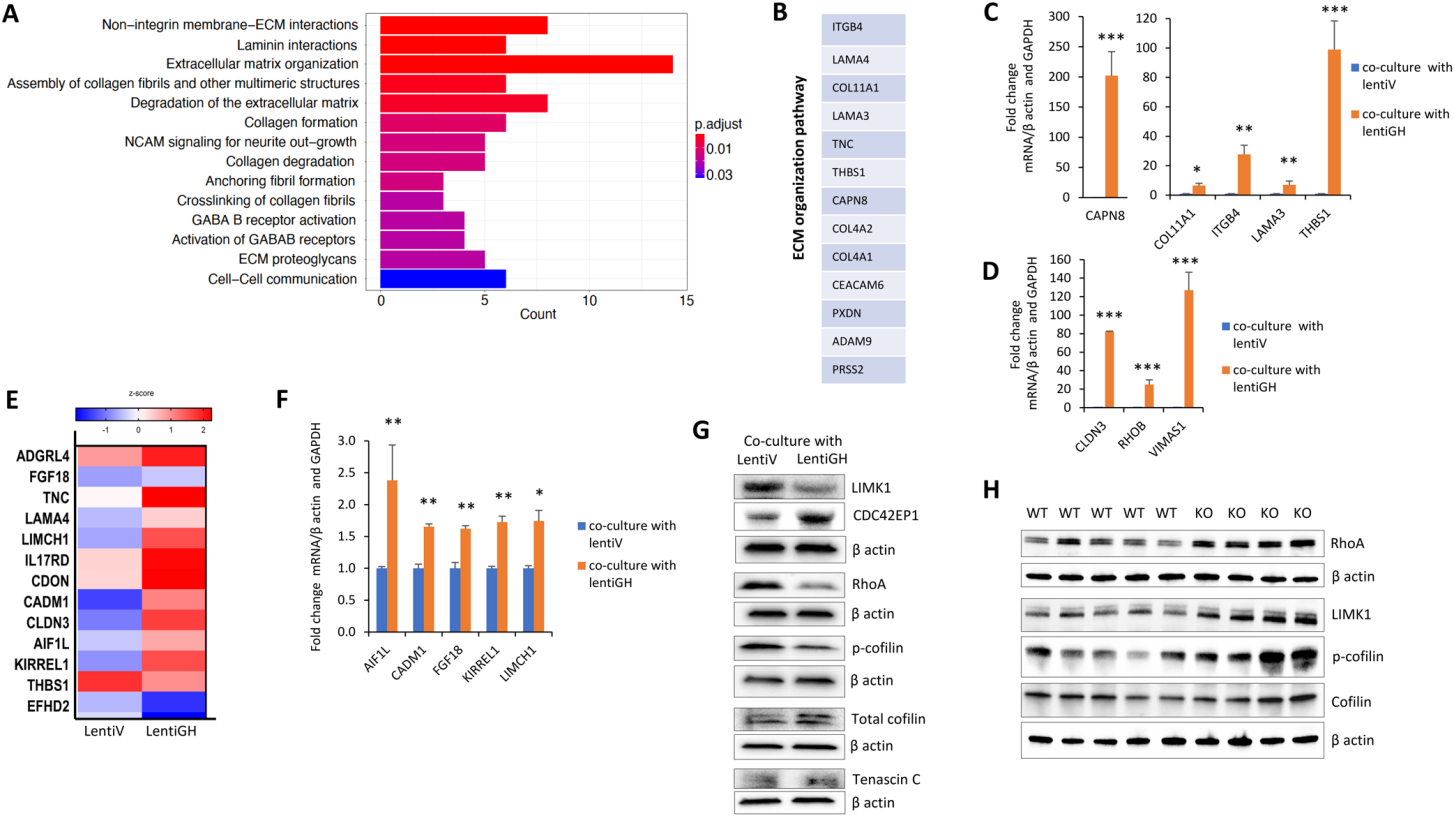
(A–D) Paracrine GH alters ECM organization, cell adhesion and cytoskeleton proteins. Three lines of organoids were infected with lentiV or lentiGH, both expressing GFP, and cultured for 5 weeks then sorted for GFP‐positive (expressing either lentiGH or lentiV) and GFP‐negative intact neighboring cells. GFP‐negative cells cocultured with GFP‐positive lentiV or lentiGH organoid cells were compared. (A) Reactome analysis of RNA‐seq results showed ECM organization pathway was most affected by paracrine GH. (B) ECM organization genes (Reactome). (C) RT‐PCR validation of ECM organization pathway genes in three organoid lines. (D) RT‐PCR validation of genes involved in ECM adhesion in three organoid lines. (E‐F) KEGG analysis of RNA‐seq results (see Figure S8B). (E) Representative color‐coded heat map reflecting fold‐change of cell adhesion gene expression between organoid cells cultured in the presence of lentiV versus cells cultured in the presence of lentiGH. (F) Validation of adhesion gene expression by RT‐PCR. Normalized averaged PCR results from three organoid lines are expressed as fold‐change versus control. In C, D, F results are depicted as mean ± SEM. *p < 0.05, **p < 0.01. Expression of CLDN3 and THBS1 is depicted in C and D. (G) Cytoskeleton proteins in organoid cells cultured in the presence of lentiV or lentiGH. (H) Cytoskeleton proteins in the colon tissue of WT and GHRKO mice. ImageJ quantification of protein expression is depicted in Figure S9A,B.

The transcriptomic landscape of organoid cells subjected to paracrine GH was assessed by bulk RNA‐seq. Differential expression analysis revealed 163 genes differentially expressed between GH and vector (FDR < 0.05) (Table S1). Over‐representation analysis identified multiple significant pathways, including collagen degradation (FDR 2.23 × 10^−2^) and laminin interactions (FDR 6.19 × 10^−4^). Over‐representation analysis using Reactome (Figure 4A), GO (Figure S8A), and KEGG tools (Figure S8B) indicated that organoid cells cultured in the presence of paracrine GH exhibit significant changes in ECM organization.

ECM interacts with cells to regulate proliferation, migration, and differentiation (Bonnans et al. 2014). GH exposure resulted in overexpressed ECM organization pathways comprising 12 genes (Bonnans et al. 2014) (Figure 4A,B), including integrin beta‐4 (ITGB4) linking the ECM to the cytoskeleton (adj *p* = 0.006), and genes encoding structural components laminin 3 (LAMA3) (adj *p* = 0.0029) and collagen type XI (COL11A1) (2.1‐fold GH, adj *p* = 3.89 × 10^−2^). CEACAM6, which interacts with ECM and promotes migration (Wu et al. 2024), CAPN8 (adj *p* = 0.003), a protease involved in ECM degradation (Song et al. 2024), and thrombospondin1 (THBS1) (*p* = 0.00041), encoding a protein interacting with MMPs (Bein and Simons 2000), all were significantly upregulated, and gene expression changes were validated by RT‐PCR (Figure 4C).

Additionally, we found that genes regulating ECM adhesion were induced by GH exposure, including the noncoding antisense transcript vimentin antisense RNA 1 (VIM‐AS1) (Santos et al. 2022), claudin 3 (CLDN3) encoding a protein regulating tight junctions (Morita et al. 1999), and Ras homolog gene family member B (RhoB) encoding a key regulator of cell migration (Warner et al. 2019). All are also involved in cell‐to‐cell adhesion and all assessed by RT‐PCR (Figure 4D). Induced MMP2 and MMP9 mRNA levels (Figure 2H) suggestive of decreased attachment to ECM and support these observations.

KEGG analysis also identified two additional pathways significantly affected by paracrine GH: the phosphatidylinositol 3‐kinase (PI3K)/Akt pathway described previously (Diaz et al. 2012; Gong et al. 2020), and the focal adhesion pathway (*p* = 0.016) (Figure S8B). Paracrine GH significantly altered newly observed genes involved in cell adhesion and cytoskeleton rearrangements (Figure 4E). We validated increased expression of allograft inflammatory factor 1 (AIF1L) (De Leon‐Oliva et al. 2023), cell adhesion molecule (CADM1) (Li et al. 2021), KIRREL 1 (Paul et al. 2022), and Lim and calponin homology domain 1 (LIMCH1) (Alifanov et al. 2022), and found FGF18 (Tsuchiya et al. 2023), an ECM component, is also induced by GH (Figure 4E,F). Functionally, many genes involved in this pathway intercross with genes implicated in the ECM structure pathway, including COL4A1, ITGB4, LAMA3, CLDN3, and THBS1 (Figure 4B–D).

Based on these results, it appears likely that changes in ECM, focal adhesion, and cytoskeletal rearrangement underlie paracrine GH actions on the observed cell motility (Figure 2D,E, Figure 2I,J, and Figure 3C).

### 2.7 Paracrine GH Disrupts Downstream Cytoskeleton Protein Expression

Given the above findings, we next evaluated whether changes in gene expression observed in organoid cells exposed to paracrine GH translate into downstream changes in proteins involved in cell‐ECM communication and cell motility.

Cofilin, required for actin filament dynamics and cell migration, is phosphorylated and inactivated by LIM kinase 1 (LIMK1) (Scott and Olson 2007; Villalonga et al. 2023). In cells growing in the presence of paracrine GH, we found LIMK1 significantly suppressed, with consequent decreased phospho‐cofilin and increased total cofilin expression associated with cell migration (Mizuno 2013). Consistent with these results, CDC42EP1, involved in filopodia formation, actin polarization, and cell migration (Farrugia and Calvo 2016; Hirsch et al. 2001) was also induced (Figure 4G and Figure S9A). By contrast, in GHRKO mice devoid of GHR signaling, LIMK1 and phospho‐cofilin were induced, indicative of decreased cofilin activity (Figure 4H and Figure S9B).

RhoA, which regulates ECM integrity and cell–cell adhesions (Arthur and Burridge 2001), was suppressed by paracrine GH (Figure 4G and Figure S9A), while it was upregulated in the colon of GHRKO mice (Figure 4H and Figure S9B). Notably, GH exposure induced the ECM glycoprotein tenascin C, indicative of weakened focal adhesion and increased motility (Chiquet‐Ehrismann and Tucker 2011), concordant with induced FGF18 (Figure 3B and Figure S6A).

These results indicate that paracrine GH regulates the expression of ECM, focal adhesion, and cytoskeleton genes manifested as cytoskeletal changes controlling cell motility.

### 2.8 Paracrine GH Alters Phosphorylation of Cytoskeletal and Cell Migration Proteins

Protein phosphorylation and dephosphorylation may regulate proliferation, migration, and invasion. Phosphorylation often occurs quickly, and may result in transient functional effects. We therefore chose to test stable phosphorylation that potentially may result in functional changes of the proteins (Ardito et al. 2017). Mimicking paracrine GH effects, we treated hNCC with 500 ng/mL GH for 6 and 24 h and performed unbiased quantitative phosphoproteomic and proteomic analyses to determine changes in cell phosphorylation status. Overall, we reproducibly quantified 15,657 phosphopeptides on 3603 proteins and 17,766 phosphopeptides on 4241 proteins in two phosphoproteomic analyses, respectively, as well as 6841 proteins in proteomic analysis (Table S2). Although the abundance of most proteins did not change, 1.2% of all proteins quantified were significantly increased and 2.4% decreased in cells treated with GH for 6 h. In the phosphorylation analysis, 2.6% and 2.5% of phosphopeptides were significantly increased and 1.2% and 1.6% decreased in GH‐treated cells in the two phosphoproteomic analyses, respectively (Table S2).

**FIGURE 5:**
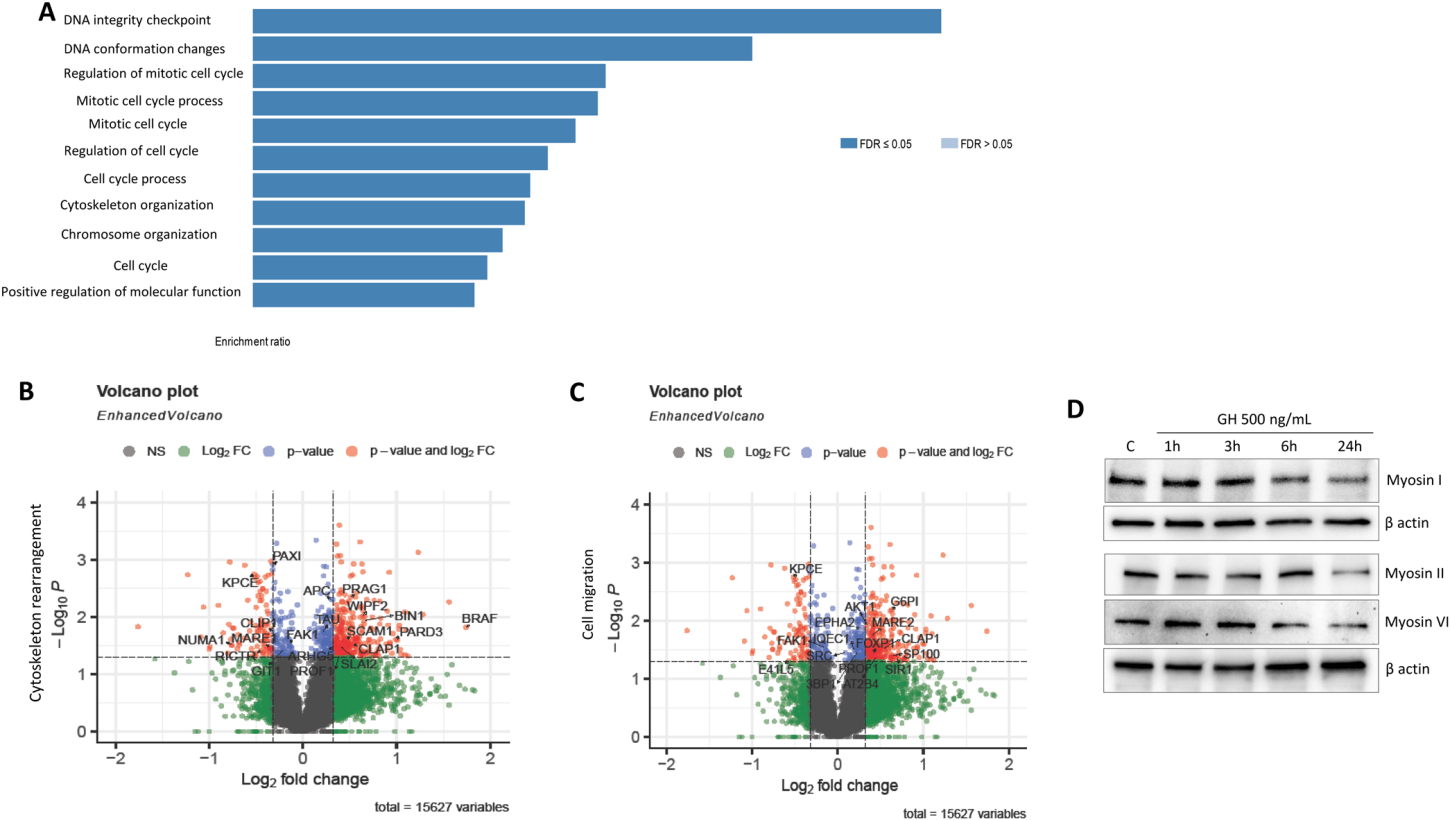
Paracrine GH alters phosphorylation of cytoskeleton and cell migration proteins and suppresses myosin expression. hNCC were treated with 500 ng/mL GH then analyzed for phosphoproteomic changes. Depicted results are from three independent biological replicates. (A) Over‐representation analysis of human phenotype ontology database depicting pathway changes after 6 h of treatment. (B, C) Volcano plots after 24 h of treatment depicting log2 ratios of peptides that belong to (B) cytoskeleton rearrangement and (C) cell migration pathways, plotted against the negative log10 of the *p* value of their fold change. Peptides above −log10 = 1.35 (shown in red) corresponding to *p* < 0.05 are considered statistically significant. (D) Myosin expression 24 h after treatment with GH. ImageJ quantification of protein is depicted in Figure S9C.

GO and pathway analysis was performed on proteins with phosphorylation sites that significantly increased or decreased in WebGestalt (Liao et al. 2019). After 6 h of treatment, pathways involved in regulation of the cell cycle (*p* = 5.9423 × 10^−7^) and cell cycle processes (*p* = 5.4508 × 10^−7^), cytoskeleton organization (*p* = 8.7270 × 10^−7^), and DNA integrity checkpoints (*p* = 0.00015556) were the most altered processes (Figure 5A and Table S2), consistent with results obtained by RNA‐seq (Figure 4A–F) and also with our previous studies showing DNA damage accumulation due to impaired DNA repair (Chesnokova et al. 2021; Chesnokova, Zonis, Barrett, Kameda, et al. 2019). After 24 h, we observed significant cytoskeleton and cell migration pathway changes (Figure 5B,C and Tables S2 and S3). These included significant changes in BIN1, which regulates actin‐membrane interaction (Picas et al. 2024); CLIP1, which regulates cytoskeletal dynamics; PROF1 (profilin1), an actin sequestering protein; AKT and CLAP1, involved in both cytoskeleton rearrangement and cell migration pathways (Islam et al. 2023; Zhou et al. 2014); and FOXP1, which regulates cell migration (Oskay Halacli 2017).

Using proteomic analyses, we found ubiquitously decreased expression of myosin family proteins (*p* = 0.05) and phosphopeptides (*p* < 0.05) (Table S4), (Hartman and Spudich 2012). In cells treated with GH, Western blotting results affirmed time‐dependent decreased abundance of myosin I, II, and VI, all involved in cytoskeleton rearrangement and motility (Figure 5D and Figure S9C).

### 2.9 Paracrine npGH Alters Chromosomal Stability

**FIGURE 6:**
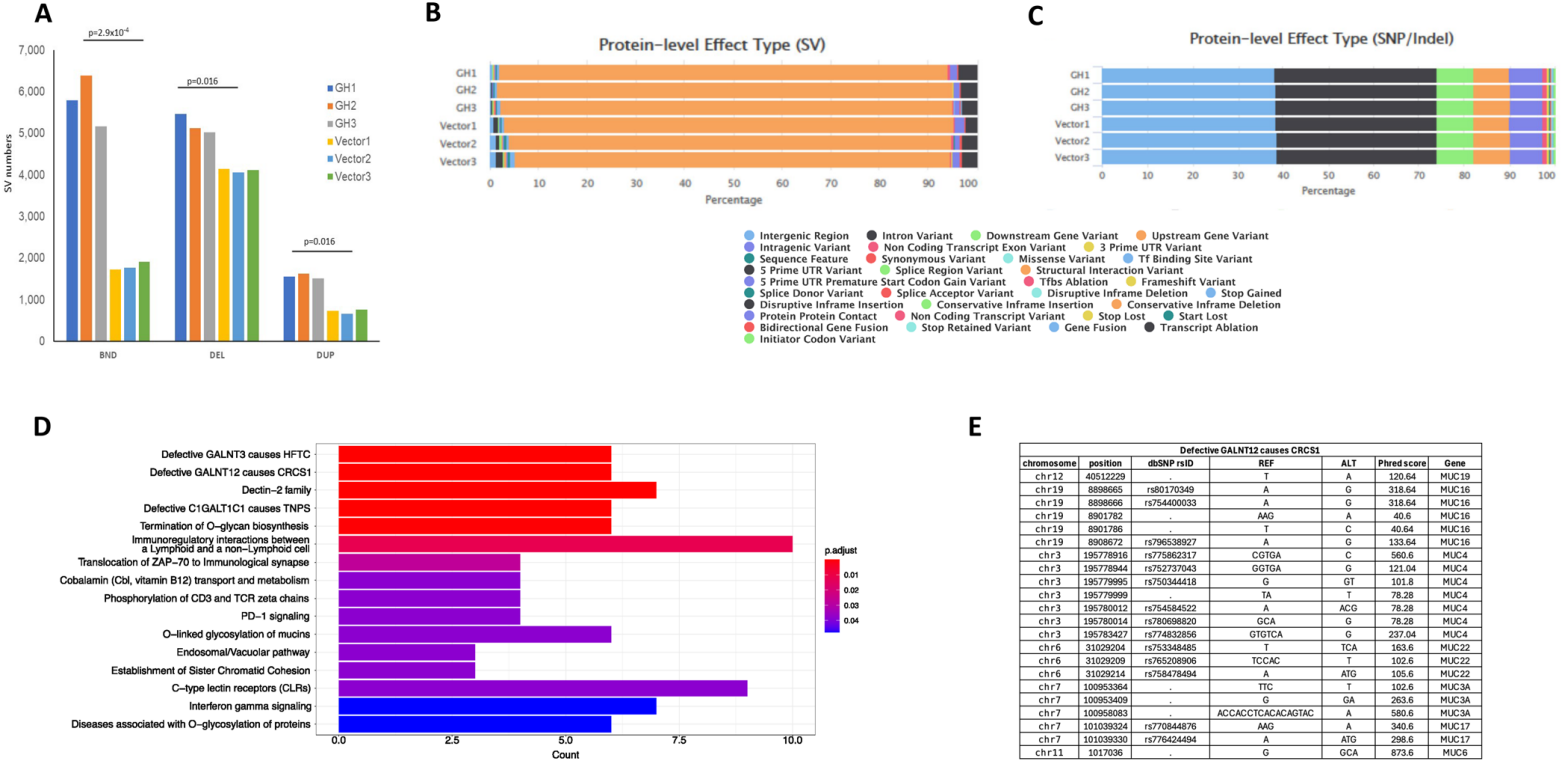
Paracrine GH induces chromosomal instability increasing structural variants. The experiment is described in Figure 4. (A) Bar charts of SV type across GH‐affected (GH, *n* = 3) and vector‐affected (*n* = 3) samples after whole‐genome sequencing. BND, breakend; DEL, deletion; DUP, duplication. (B) Distribution of protein‐level effect types for SV. (C) Distribution of protein‐level effect types for SNP/Indels. (D) Selected GH‐unique high‐impact variants subtypes with pathway enrichment analysis of 679 genes. (E) Mucin genes from “Defective GALNT12 causes CRCS1” pathway with genomic position of SNP/Indel, dbSNP id where given, associated gene, and Phred score.

Comprehensive genomic profiling was undertaken to define the molecular landscape of organoids cultured in the presence of paracrine GH. We tested whether npGH impacts chromosomal stability, a determinant of age‐associated changes (Lopez‐Otin et al. 2023). Whole‐genome sequencing (WGS) was performed in organoids derived from 3 independent iPSC lines infected with lentiGH or lentiV. Large structural variations (SV) were observed, including duplications (DUP), deletions > 50 bp (DEL) and breakend variants (BND). The mean whole‐genome SV load was significantly increased, showing 15,132 (SD 804) for GH and 9511 (SD 338) for control. Increases in DUP (adj *p* = 2.7 × 10^−4^), DEL (adj *p* = 1.7 × 10^−2^), and BND (adj p = 1.7 × 10^−2^) in GH‐exposed as compared to control cells were observed (Figure 6A). At the protein‐level effects of variants, the largest proportional changes were ascribed to increased structural interaction variants changing from an average of 90.8% of all SV in controls to 92.8% of all SV in GH‐exposed cells, indicating a disruption of internal polypeptide structure interactions. Concomitant decreased relative proportions of SV were observed for GH‐exposed cells relating to intergenic regions (1.2% control vs. 0.4% GH) and intron variants (1.0% control vs. 0.3% GH), albeit with similar absolute numbers compared to controls, indicating that GH has little effect on regulatory elements and splicing events (Figure 6B).

Short variant discovery for single nucleotide polymorphisms and insertions/deletions (SNP/Indels) was performed using GATK. Deconvolution of variant number by genomic region showed that the only significant difference between GH‐exposed and control cells was seen in exon regions, with a reduction from an average of 83,519 SNP/Indels in GH‐exposed cells to 82,399 SNP/Indels in controls (*p* = 4.86 × 10^−3^). At the protein level, SNP/Indels were predominantly annotated as either intergenic regions or intron variants (> 70% in all samples) (Figure 6C). We identified 679 “high impact” mutations unique to the GH group, including stop‐gain nonsense mutations, frameshift variants, splicing variants affecting splice donor or acceptor sites, which are critical for intron removal during splicing, and initiator codon variants, which have a functional impact on encoded proteins (Figure 6D). Pathway enrichment analysis of these GH‐specific mutations identified pathways including “O‐linked glycosylation of mucins” (adj *p* < 0.05) and “Defective GALNT12 causes CRCS1” (adj *p* < 0.05), which includes MUC16/MUC19 with splice region variants and MUC3A/MUC4/MUC6/MUC17/MUC22 with frameshift variations (Figure 6E).

## 3 Discussion

We assessed paracrine and autocrine consequences of age‐induced local npGH induction on normal human colon epithelial cells. Although the role of the microenvironment in tumor progression and metastatic conversion has been well studied (Campbell and Booth 2023; de Visser and Joyce 2023; Fane and Weeraratna 2020), little attention has been given to elucidating characteristics of aging‐associated changes in the nontumorous tissue microenvironment, on normal tissue cell homeostasis, and on early age‐associated microenvironment changes.

After confirming here that npGH expression increases with age in both epithelial and stromal cells (Chesnokova et al. 2021) and that GH is expressed in senescent human colon cells, we went on to elucidate mechanisms for autocrine/paracrine GH action. Autocrine GH action on breast tumor cell migration and invasion (Mukhina et al. 2004), and on nontumorous endothelial cell migration (Messias de Lima et al. 2017), macrophages (Dos Santos Reis et al. 2018), keratinocytes (Lee et al. 2010), and cornea epithelial cells (Ding et al. 2015) has been reported. We have reported that 2 ng/mL GH secreted by senescent hNCC results in increased proliferation and neighboring cell transformation (Chesnokova et al. 2024). Our results now further show that local autocrine and paracrine GH induce neighboring normal epithelial cell migration. Although in our current experiments, cells infected with lentiGH secreted up to 120–700 ng/mL in culture medium depending on the cell type, it is likely that persistent secretion of even lower amounts of GH from senescent cells in vivo will result in similar changes in neighboring cells.

EMT leads to the acquisition of a mesenchymal phenotype enabling motility by epithelial cells losing apical‐basal polarity, rearranging the cytoskeleton, and reducing cell–cell adhesive properties. EMT transcription factors including Snai1, Twist1/2, and Zeb1/2 evoke EMT in part through repression of E‐cadherin (Brabletz et al. 2021; Yang et al. 2020), leading to nuclear β‐catenin translocation, facilitating the expression of cMyc and other genes involved in proliferation, migration, and cell‐matrix interactions (Cadigan and Waterman 2012; Miller et al. 1999). Cytoplasmic APC, a component of the distraction complex, limits nuclear β‐catenin proproliferative and protumorigenic activity (Clevers and Nusse 2012). In our experiments, it is likely that the increased migration and invasion we observed in cells overexpressing GH or exposed to paracrine GH occur as a consequence of induced EMT. Exogenous GH activation of EMT was shown earlier (Basu et al. 2017; Chesnokova et al. 2024, 2016; Kopchick et al. 2022; Qian et al. 2020). Here, we now elucidate mechanisms underlying local GH effects on neighboring cell EMT. Local autocrine and paracrine GH activate Twist2, suppressing E‐cadherin and APC, and constraining nuclear β‐catenin translocation. Nuclear β‐catenin binds TCF/LEF and induces cMyc (Das et al. 2023) and the transcription of MMP2/9 involved in ECM remodeling (Chang and Werb 2001). These events support a mechanism whereby GH induces migration and invasion in normal human epithelial cells.

Effects of autocrine/paracrine GH on EMT transcription factors and E‐cadherin in these normal cells are similar to reported GH action in the tumor microenvironment (Basu et al. 2025). In addition, in our experiments, paracrine GH is shown to deregulate expression of multiple genes and proteins that regulate cell polarization and cell adhesion, increasing epithelial cell motility. The colonic mucosal lining regenerates every 3–5 days, being renewed by continued epithelial cell migration from crypts along the villi (van der Flier and Clevers 2009). However, extensive and deregulated epithelial migration due to altered intercellular adhesions may lead to neoplastic transformation (Burclaff and Mills 2018; Furuse 2010; Hartsock and Nelson 2008).

Importantly, EMT is only partially activated in normal nonneoplastic epithelial cells, which likely reside in an intermediate stage retaining both epithelial and mesenchymal markers. This hybrid EMT has tumor‐initiation capacity, increases cell migration, and enables a cell survival advantage (Brabletz et al. 2021). In addition, EMT transcription factor activation is required for fibrosis development (Yang et al. 2020), which increases with age (Ferrucci and Fabbri 2018; Martinez et al. 2017). For example, sustained FGF18 upregulation, also observed in our experiments, induces collagen and ECM, ultimately promoting liver fibrosis (Tsuchiya et al. 2023). A potential role for endocrine GH in fibrosis development has been reviewed in detail (Kopchick et al. 2022). Our results now suggest that local npGH, by triggering EMT, modifies the microenvironment, favoring age‐associated neoplastic growth and fibrosis.

EMT activates cell motility, involving ECM remodeling. ECM components serve as ligands for cell surface integrins that regulate adhesion, proliferation, migration, survival, and differentiation (Bonnans et al. 2014), while the loss of tissue ECM integrity may be associated with neoplasia (Hanahan and Weinberg 2011).

Using RNA‐seq of intact organoids cocultured with organoids infected with lentiGH as well as by Reactome‐ and enriched GO analysis, we show that pathways mostly affected by local paracrine GH are ECM‐associated. Validated by RT‐PCR, we found that several genes involved in this pathway are upregulated by paracrine GH action, including glycoproteins LAMA3 and THBS1, both of which act as integrin ligands. Furthermore, by regulating ECM LAMA3 (Qin et al. 2024), calpain 8 (CAPN8) (Song et al. 2024), ITGB4 (S. W. Lee et al. 2010), and CEACAM6 (Lewis‐Wambi et al. 2008), paracrine GH appears to modulate cell adhesion and motility. In support of this finding, we also show in organoid cells cultured in the presence of paracrine GH induction of tenascin C, an ECM glycoprotein activated in malignancies and in modulating adhesion and motility, indicative of ECM reorganization (Midwood et al. 2011; Spenle et al. 2015). Our observed induction of MMP2/9 also points to ECM rearrangements in the presence of GH, as dysregulated MMP activity is associated with cancer, fibrosis, and cardiovascular disease (Lampi and Reinhart‐King 2018). The observed induction of other genes from this pathway, including VIM‐AS1 (Beltran et al. 2008), CEACAM6 cell adhesion molecule (Wu et al. 2024), and CLDN3, a part of the tight junction protein family (Heiskala et al. 2001), are consistent with activated EMT.

Cell migration initiates with the protrusion of the cell membrane, followed by the formation of new adhesions (Parsons et al. 2010). KEGG analysis, while confirming GH involvement in the ECM‐receptor interaction pathway (Figure 6), also indicated significant changes in focal adhesion. We validated the upregulation of genes involved in cell‐to‐cell and cell‐to‐ECM adhesion, including AIF1L (Yasuda‐Yamahara et al. 2018), CADM1 (H. Li et al. 2021), KIRREL1 (Paul et al. 2022), and LIMCH1 (Alifanov et al. 2022), all of which regulate focal adhesion assembly.

Endocrine GH induces actin stress fiber depolymerization (Goh et al. 1997), and generation of membrane ruffles is required for cell migration (Herrington et al. 2000). In our experiments, we observed altered gene expression by local paracrine GH that translated to in vitro and in vivo changes in cytoskeleton protein expression. In organoid cells growing in the presence of GH‐secreting cells, CDC42 effector protein CDC42EP1 is induced while RhoA is significantly downregulated, indicative of dysregulated cytoskeleton organization. The Rho family, including Rho, Rac, and CDC42, regulates pathways activated during cell migration (Clayton and Ridley 2020). Thus, CDC42 activation is required to establish filopodia at the leading edge of migration (Nobes and Hall 1995) and RhoA regulates the actin cytoskeleton, including cell morphology, polarity, adhesion, migration/motility, and protrusion (Etienne‐Manneville and Hall 2002; Nobes and Hall 1999; Ridley et al. 2003). Functionally, some genes involved in the focal adhesion pathway intercross with those implicated in the ECM structure pathway. Thus, RhoA also mediates ECM protein expression (Plotnikov et al. 2012).

LIMK1 phosphorylates and inactivates cofilin (Arber et al. 1998; Bernstein and Bamburg 2010; Villalonga et al. 2023); LIMK1, a downstream effector of Rho GTPases, is involved in cytoskeletal remodeling of both actin filaments and microtubules. Actin polymerization at the barbed ends of the lamellipodium pushes the membrane forward (Pollard and Borisy 2003), while cofilin regulates lamellipodial protrusion by stimulating actin filament disassembly, thereby supplying actin monomers for polymerization (Kiuchi et al. 2011). In our experiments, LIMK1 was suppressed in organoids exposed to paracrine GH, likely a consequence of observed decreased RhoA (Kiuchi et al. 2011) and LIMK1 suppression in the face of total (active) cofilin increase. By contrast, GHRKO mice with disrupted GH signaling exhibited increased LIMK1, RhoA, and phospho‐cofilin as well as decreased cofilin/phospho‐cofilin ratio, likely indicative of decreased potential for pathological colon cell motility in animals deprived of GH signaling. These alterations may partially explain low rates of spontaneous tumors in GHRKO mice (Junnila et al. 2013; List et al. 2011), lower rates of intestine and colon tumors in hybrid *APC* ^*min+/−*^ GHRKO mice (Chesnokova et al. 2016), and low cancer rates in people harboring GHR disruptive mutations (Guevara‐Aguirre et al. 2011).

In addition to its effects on the Jak/STAT pathway, GH phosphorylates or dephosphorylates multiple signaling proteins including Ras/Raf/MAPK kinase (MEK)1/Erk1/2 (Vanderkuur et al. 1997; Wang et al. 2009), PI3K/Akt/mTORC1 (Argetsinger et al. 1995; Costoya et al. 1999; Hayashi and Proud 2007; Ridderstrale et al. 1995), and C/EBPβ (Piwien‐Pilipuk et al. 2001). GH also affects the phosphorylation of p130Cas/Bcar1 associated with the cytoskeleton (Zhu et al. 1998), and short‐term GH treatment (5–15 min) of 3T3‐F442A preadipocytes affected the phosphorylation of actin cytoskeleton and focal adhesion pathway proteins (Ray et al. 2012).

Exposure to paracrine GH for 6 h mostly affected phosphoproteins belonging to cytoskeleton organization, chromosomal organization, and cell cycle regulation pathways, supporting our finding that GH alters focal adhesion pathway gene expression. After 24 h of paracrine GH exposure, we observed significant changes in the phosphorylation of proteins involved in cytoskeleton rearrangement and cell motility pathways.

Proteomic analysis revealed that GH downregulates myosin family proteins, which move cargo along actin tracks. Nonmuscle myosin IIA is involved in unfolding and activating motor proteins regulating transcription and gene expression (Porro et al. 2021; Shahid‐Fuente and Toseland 2023) and cell polarity (Juanes‐Garcia et al. 2015). Myosin VI functions in vesicular membrane traffic, cell migration, and mitosis (Buss et al. 2004; Magistrati and Polo 2021; Shahid‐Fuente and Toseland 2023). Myosin 1β (nuclear myosin) contributes to genome integrity and facilitates chromosomal rearrangement and DNA damage repair (Cook et al. 2020; Venit, Mahmood, et al. 2020; Venit, Semesta, et al. 2020). Myosin 1β suppression by GH may also contribute to chromosomal instability observed here, as well as to suppressed DNA repair (Chesnokova et al. 2024).

Maintenance of genomic stability is required for normal cell functioning (Hosea et al. 2024). In normal cells, chromosomal instability arises from unrepaired DNA damage and chromosomal mis‐segregation leading to genomic instability, a hallmark of aging (Lopez‐Otin et al. 2013). SV reflects DNA damage and genomic instability and occurs in both normal tissue and cancers (Colnaghi et al. 2011; Li et al. 2020; Sharma et al. 2016). Our results show significantly increased numbers of SV in organoid cells exposed to paracrine GH including duplications, deletions, and breakends. An important mechanism of SV generation is deficient nonhomologous end joining (NHEJ) repair. It is plausible that the impact of GH action emanates from insufficient DNA damage repair, as we previously showed that both endocrine and local GH suppressed NHEJ DNA repair pathways in normal human colon cells, resulting in DNA damage accumulation (Chesnokova et al. 2021; Chesnokova, Zonis, Barrett, Kameda, et al. 2019). Moreover, after infecting organoids with lentiGH, we found that uninfected cells exposed to paracrine GH exhibit suppressed DNA repair with increased DNA damage (Chesnokova et al. 2024). With age, DNA damage response activity is reduced (Feng et al. 2007; Gutierrez‐Martinez et al. 2018), and induced local GH may play a role in this decrease. Other DNA repair‐related factors, such as Fanconi anemia proteins, are also important for preventing chromosomal instability (Hosea et al. 2024; Naim and Rosselli 2009), and this pathway was significantly affected by GH in somatotroph pituitary tumors that express excess GH and exhibit high levels of SV (Ben‐Shlomo et al. 2020). Importantly, GH suppresses p53 in DNA‐damaged cells (Chesnokova et al. 2013, 2016; Chesnokova, Zonis, Barrett, Kameda, et al. 2019), thereby providing a prolifrative advantage to cells with chromosomal instability (Hosea et al. 2024).

Our studies also revealed specific mutations attributed to paracrine GH in epithelial cells. Pathway enrichment analysis identified “O‐linked glycosylation of mucins” (adj *p* < 0.05) and “Defective GALNT12 causes CRCS1” (adj *p* < 0.05) pathways, specifically including the genes MUC16/MUC19 with splice region variants and MUC3A/MUC4/MUC6/MUC17/MUC22 with frameshift variations (Figure 3E). Interestingly, GALNT12 is associated with colorectal and glioma malignancies (Evans et al. 2018). Initiation of mucin‐type O‐linked glycosylation is governed by the GALNT family (Brockhausen et al. 2009), and glycosylation is required for adhesion, migration, and immune surveillance (Ohtsubo and Marth 2006). PpGalNAc‐T12, encoded by GALNT12, is highly expressed in normal colon tissue and downregulated in colonic cancers (Guo et al. 2004, 2002). Aberrant glycosylation is a hallmark of colorectal cancer (Bergstrom and Xia 2013; Brockhausen 2006) and defects in the O‐linked pathway may contribute to colorectal pathogenesis (Brockhausen 2006; Brockhausen et al. 2009; Tran and Ten Hagen 2013). Although we demonstrate GH actions in normal nontumorous tissue, it is likely that similar mechanisms, including ECM restructuring, cytoskeleton rearrangement, chromosomal instability, and increased number of mutations, are at play in the tumor microenvironment where GH is abundantly expressed.

Effects of endocrine GH on aging have been reviewed extensively. GH excess results in premature aging, fibrosis, and tumorigenesis in GH‐expressing transgenic mice and in acromegaly patients (Aguiar‐Oliveira and Bartke 2019; Anisimov and Bartke 2013; Bartke 2008, 2021; Colon et al. 2019; Corpas et al. 1993). Disrupted GH/IGF1 signaling extends lifespan and reduces age‐associated pathologies in experimental models (Anisimov and Bartke 2013; Bartke 2021; Basu et al. 2018; List et al. 2024; Qian et al. 2022), and also prevents cancer in human subjects (Aguiar‐Oliveira and Bartke 2019). Although circulating GH decreases with age, npGH expression increases with aging in colon tissue, and we now portray a broad mechanistic picture of local GH action on the normal aging microenvironment landscape. The multistage model of carcinogenesis from tumor initiation to promotion and then progression requires incorporation of aging‐dependent somatic changes defined by the microenvironment (Fane and Weeraratna 2020; Rozhok and DeGregori 2019). As cancer risk increases with age, it is likely that key factors associated with aging play a role, including increased genomic damage accumulation. Our results offer insight into how local GH, induced in response to DNA damage or/and senescence in aging tissue, regulates ECM, cytoskeleton proteins, and phosphorylation levels, promoting changes that may alter tissue integrity and lead to age‐associated pathologies including neoplasms.

## 4 Limitations of the Study

The tissue microenvironment comprises fibroblasts, endothelial cells, pericytes, adipocytes, ECM, and immune cells. As autocrine/paracrine GH is induced with DNA damage in aging, it is important to examine its effects on the tissue microenvironment during aging. For example, we showed that GH is upregulated in stromal fibroblasts (Chesnokova et al. 2021) and in intestinal immune cells in patients with inflammatory bowel disease (Chesnokova et al. 2016). Accordingly, GH could alter the tissue microenvironment by disrupting proliferation and differentiation of intestinal stem cells (Chen et al. 2018) or stromal macrophage and endothelial cells (Chesnokova and Melmed 2023).

The question remains as to what extent the effect of local GH is mediated by IGF1. Several comprehensive studies have shown that direct reduction of GH rather than secondary reduction via IGF1 benefits aging in mammalian systems, likely through reduced inflammation and insulin sensitivity (Brown‐Borg 2022). We recently showed that in the colon GH acts directly, independent of IGF1, to induce DNA damage (Chesnokova, Zonis, Barrett, Gleeson, and Melmed 2019). However, in cells and tissues where GH activates IGF1, IGF1 may counteract GH action on genome stability, inducing DNA repair and decreasing radio‐ or chemotherapy sensitivity, especially as the IGF1 receptor also interacts with the GH receptor and may augment GH signaling (Gan et al. 2014). Furthermore, IGF1 receptor depletion impairs ATM kinase activity in murine melanoma cells and enhances radiosensitivity of prostate cancer cells (Chitnis et al. 2014; Turney et al. 2012). Therefore, we cannot exclude that, in vivo, GH and IGF1 act concomitantly to induce DNA damage and chromosomal instability. If so, beneficial effects of GH deficiency on health‐ and lifespan may also be mediated, at least in part, by decreased IGF1.

## 5 Materials and Methods

### 5.1 Mice

Athymic nude male Nu/J and GHRKO mice were purchased from The Jackson Laboratory. As breeding was undertaken with heterozygous males and females, WT and GHRKO mice were obtained from the same breeding. Heterozygous mice were backcrossed with WT mice at least 6 times. Three‐month‐old males were used for experiments.

### 5.2 3D Human Intestinal Organoids

Three‐dimensional intestinal organoids from fibroblast‐derived iPSCs were generated using episomal plasmid reprogramming and cultured as described (Barrett et al. 2014; Workman et al. 2018). To induce definitive endoderm formation, iPSCs were cultured with activin A (100 ng/mL; R&D Systems) with increasing concentrations of FBS (0%, 0.2%, and 2% [vol/vol] on days 1, 2, and 3, respectively). Wnt3A (25 ng/mL; R&D Systems) was also added on the first day of endoderm differentiation. To induce hindgut formation, cells were cultured in Advanced DMEM/F12 with 2% (vol/vol) FBS along with CHIR 99021 (2 μM; Tocris) and FGF4 (500 ng/mL; R&D Systems). After 3–4 days, free‐floating epithelial spheres and loosely attached epithelial tubes were harvested. Epithelial structures were subsequently suspended in Matrigel (Corning, cat#354248) and overlaid in intestinal medium containing CHIR99021 (2 μM; Tocris), noggin, and EGF (both 100 ng/mL; R&D Systems), and B27 (1×; Invitrogen). Organoids were sorted for EPCAM, an epithelial cell marker, 30 days after differentiation and passaged every 7–10 days thereafter. Intestinal organoids were validated after identifying enterocytes, goblet cells, Paneth cells, and enteroendocrine cells as well as the expression of CDX2 (Gao et al. 2009).

### 5.3 Cells and Treatments

hNCC line #1 and line #2 (Applied Biological Materials, lot #HC1211 and lot #0145834955002, respectively) were obtained from deidentified normal colon sections of two individuals and validated as cytokeratin 18‐ and 19‐expressing colon epithelial cells. Donor sex and age were not available. Cells were cultured in PriGrow III Medium (Applied Biological Materials) supplemented with 5% FBS and with antibiotic/antimycotic solution (Gemini Bio‐Products), then infected or treated before passage 5. Except as specifically mentioned, all experiments were conducted in hNCC line #1.

hCF (Cell Biologics) were cultured in Fibroblast Medium with Supplement (Cell Biologics, cat#M2267). Cells from passage 3 were used for experiments. HCT116 cells were cultured in McCoy 5A medium with 10% FBS.

For GH treatment, hNCC were plated into 6‐well plates in PriGrow III Medium in triplicates. The following day, medium was replaced with PriGrow III Medium containing 0.1% BSA without serum, and 500 ng/mL recombinant human GH (BioVision, cat#4769) was added for 24 h. The following day, GH was added for shorter time periods as indicated for the experiment, and all cells were collected simultaneously.

### 5.4 Mouse Xenografts

HCT116 cells infected with lenti‐mGH or lentiV (5 × 10^5^ cells in 0.05 mL PBS) were mixed (1:1) with High Concentration Matrigel Matrix (Corning, cat#354263) and injected into the right flank of nude athymic male mice subcutaneously to establish a model of excess systemic GH. Mice were sacrificed 5 weeks after injection. Circulating levels of GH were measured with Mouse/Rat Growth Hormone ELISA (ALPCO, cat#22‐GHOMS‐E01) and murine IGF1 was measured with mouse/rat IGF1 ELISA kit (ALPCO, cat#22‐IG1MS‐E01) as described (Chesnokova et al. 2016).

### 5.5 Constructs and Transfections

Lentiviral particles expressing hGH (pLV‐EF1p‐hGH1‐IRES‐eGFP‐WPRE) and respective control lentiviral particles (pLV‐EF1p‐mCherry‐IRES‐eGFP‐WPRE), as well as murine GH (EF1‐luc2‐GH‐Ubic) and vector (EF1‐luc2‐Ubic) were generated at the Regenerative Medicine Institute at Cedars‐Sinai.

hNCC or HCT116 or human colon fibroblasts were plated 1 day before transfections and infected with 50 MOI lentivirus particles with 8 μg/mL polybrene added.

Organoids were dispersed into a single‐cell suspension using TrypLE (Gibco, cat#12604) and mixed with Matrigel together with 50 MOI lentiviral particles and 8 μg/mL polybrene. Cells in the Matrigel bubble were plated into 24‐well plates and overlaid with intestinal organoid medium enriched with 10 nM Rock inhibitor (Tocris, cat#1254), 500 nM A8301 (Tocris, cat#2939), and 10 μM SB202190 (Tocris, cat#1254).

M50 Super 8xTOPFlash and M51 Super 8xFOPFlash (TOPFlash mutant) plasmids were a gift from Dr. Randall Moon (Addgene, plasmid #12456 and plasmid #12457).

1 × 10^6^ hNCC lentiGH or lentiV cells were nucleofected with 4 μg of M50 or M51 plasmid together with 0.1 μg pRL‐TK Renilla Luciferase Control Reporter Vector (Promega Corporation, cat#E2241) using Amaxa nucleofector I (Lonza) and Amaxa Basic Nucleofector Kit for Primary Mammalian Epithelial Cells (Lonza), program W‐01. After nucleofection, cells were plated into 4 wells of a 24‐well plate pretreated with ECL extracellular matrix from Millipore (cat#08–110). After 24 h, the luciferase assay was performed using the Dual‐Luciferase Reporter Assay System from Promega (cat, # E1960) according to the manufacturer's instructions.

### 5.6 Cocultures

For coculture experiments using hNCC line#1, hNCC line#2, or intestinal organoids, cells were infected with lentiGH or lentiV, both of which contained GFP. Infection efficiency was 40%–60%. After 1 month in culture, cells were sorted for GFP positivity using a FACSAria III cells sorter (BD Biosciences). Only GFP‐negative cells (not expressing GH or vector but growing in close proximity with GFP‐expressing cells) were included in testing.

For coculture of hNCC with normal human colon fibroblasts infected with lentiGH or lentiV, 150,000 fibroblasts per well were plated into a 6‐well plate in full Fibroblast Medium. After 6 days, the medium was changed to 1.5 mL Prigrow III Medium, and 0.4 μm Transwell Permeable 24 mm Inserts (Costar, cat#3412) were placed into the wells. 60,000 hNCC were plated into the inserts in 1.5 mL of Prigrow III Medium. Cells were cultured for an additional 4 days, collected, and used for Western blotting.

For coculture experiments assessing cytoplasmic and nuclear cell fractions, 250,000 hNCC lentiGH or lentiV cells were plated per well in 6‐well plates in 1.5 mL of Prigrow III Medium. The next day, 0.4 um Transwell Permeable inserts were placed into wells, then 250,000 untreated hNCC cells were plated into inserts. Cells were collected after 1 week, counted, and cytoplasmic and nuclear protein fractions were isolated using NE‐PER Nuclear and Cytoplasmic Extraction Reagents (ThermoScientific, cat#78833).

In all coculture experiments, secretion of GH into medium was confirmed by hGH ELISA (ALPCO, cat#25‐HGHHU‐E01).

### 5.7 Protein Analysis

For Western blot analysis, cells were homogenized and lysed in RIPA buffer (Cell Signaling, cat#9806S) with protease inhibitors (MilliporeSigma, cat#P8340) or isolated from TRIzol (Ambion, cat#15596018) and dissolved in 1% SDS according to the protocol for RNA/DNA/protein isolation from Molecular Research Center Inc. Proteins were separated by SDS‐PAGE, electroblotted onto Trans‐Blot Turbo Transfer Pack 0.2 μm PVDF membrane (BioRad), and incubated overnight with primary antibodies, followed by corresponding secondary antibodies (Amersham). To detect low‐abundance proteins, Hikari signal enhancer kit was used (Nacalai USA; cat#NU00102).

The following primary antibodies were used: GAPDH, Lamin A/C, APC, and FGF18 from Santa Cruz Biotechnology (cat#sc‐32233, sc‐517580, sc‐393704, and sc‐393471, respectively); β‐actin from Sigma (cat#A1978); nonphospho (active) β‐catenin (S45), E‐cadherin, cMyc, and RhoA from Cell Signaling (cat#190807, #3195, #5605, and #2117, respectively); Twist2 from Santa Cruz Biotechnology (cat#sc‐81417) or from Lifespan Biosciences (cat#LS‐C416907); LIMK1, cofilin, and phospho‐cofilin (S3) from Abcam (cat#ab81046, #ab54532, and #ab12866 respectively); CDC42EP1 from MyBioSource (cat#MBS9435972); tenascin C from R&D Systems (cat#AF3358); myosin 1β (nuclear) from Millipore (cat#M3567); and myosin IIa, myosin VI, and CADM1 from Cell Signaling (cat#3403, #13592, and #30994, respectively).

### 5.8 Immunohistochemistry

Formalin‐fixed and paraffin‐embedded normal colon or hyperplastic polyp tissue were obtained from the Cedars‐Sinai Biobank. GH staining was performed using ImmPRESS Amplified Polymer Kit Peroxidase (Anti‐Rabbit) (Vector Laboratories, cat#MP‐7601) according to the manual. For fluorescent double staining for β‐gal and GH, paraffin slides were baked at 60°C for 1 h, and after deparaffinization, antigen retrieval was performed in 10 mM citric buffer. Tissues were permeabilized in 1% Triton X100 for 30 min, followed by blocking in 10% goat serum for 1 h. Tissues were stained overnight at 4°C with anti β‐gal mouse monoclonal antibodies (dilution 1:50) and anti‐hGH rabbit antibodies (dilution 1:100), both from LSBio (cat#LS‐B10989 and cat#LS‐B4199, respectively) followed by goat anti‐mouse Alexa 488 and goat anti‐rabbit Alexa 568 secondary antibodies, both from Invitrogen (cat#A32723 and #A11031, respectively). Images were taken with a Leica TCS/SP spectral confocal scanner (Leica Microsystems).

### 5.9 Immunocytochemistry

LentiGH or lentiV hNCC were plated in full Prigrow III medium on coverslips placed into wells of a 6‐well plate and covered with ECL extracellular matrix (Millipore). After 48 h in culture, cells were rinsed with PBS and fixed in 4% PFA/PBS for 20 min. Cells were permeabilized with 0.25% Triton X100 for 30 min, blocked with 10% goat serum for 1 h, then stained with nonphospho (S45) active β‐catenin antibody, dilution 1:1000 in 10% goat serum overnight at 4°C followed by goat anti‐rabbit Alexa 568 and DAPI (Sigma Aldrich, cat#10236276001) for 1 h at room temperature. Cells were imaged with a Stellaris confocal microscope from Leica Microsystems.

### 5.10 Real‐Time PCR

Total RNA was isolated with TRIzol (Ambion, cat#15596018) followed by RNAeasy mini Kit (Qiagen). After DNAse I treatment (TURBO DNA free, Ambion), cDNA was synthesized from 1 μg purified RNA by the SuperScript II First‐Strand cDNA synthesis system (ThermoFisher). Quantitative PCR was performed in 20 μL reactions using IQ SYBR Green Master Mix in the BioRad IQ5 instrument (BioRad Laboratories). The following PrimePCR Assays for human mRNA were used: actin, GAPDH, MMP2, MMP9, CAPN8, COL11A1, ITGB4, LAMA3, THBS1, CLDN3, RHOB, VIM‐AS1, AIF1L, CADM1, FGF18, KIRREL1, and LIMCH1 (all from BioRAD, cat#10025636). Experiments were performed in triplicate. Relative mRNA quantities in experimental samples were determined by CFX Maestro 1.0 software expressed in arbitrary units as fold‐difference from control.

### 5.11 Cell Migration and Invasion

For migration experiments, 100,000 lentiGH or lentiV hNCC line#1 or line#2 cells were plated in 100 μL serum‐free Prigrow III Medium with 0.1% BSA into Transwell Migration Chambers (Corning, cat#354578, pore size 8 M). For invasion experiments, 100,000 hNCC line#1 cells were plated in 100 μL serum‐free medium into Corning BioCoat Growth Factor Reduced Matrigel Invasion Chambers (cat#354483) placed into 750 μL of Prigrow III Medium. After 24 h, cells were fixed in 70% ethanol, then stained with 0.1% Crystal Violet and nonmigrated or noninvaded cells were removed with a cotton swab. Images were taken at 100× magnification (at least 6 images from each chamber), and the number of migrated or invaded cells was counted.

In coculture experiments, 60,000 hNCC (or hNCC line#2) lentiGH or lentiV cells/well were plated on the bottom of a 24‐well plate in 750 μL Prigrow III Medium. After 3 days, Transwell Migration or Invasion chambers were placed into these plates with 100,000 intact hNCC (or hNCC line#2) cells in 100 μL of serum‐free medium. Cells were analyzed after 24 h. Invasion experiments were performed on hNCC line#1 only.

In coculture experiments with hCF infected with lentiGH or lentiV, 50,000 fibroblasts were plated in 1 mL of Fibroblast Medium. After 3 days, Migration or Invasion Transwell chambers were placed into wells, and 100,000 hNCC line#1 cells were plated into these chambers in 100 μL of serum‐free Prigrow III Medium with 0.1% BSA. Cells were analyzed after 24 h.

GH in coculture medium was assessed by Human Growth Hormone ELISA kit (ALPCO, cat#25‐HGHHU‐E01). Coculture medium with hNCC line #1 lentiGH had 500–700 ng/mL of GH, medium with hNCC line #2 lentiGH had 300–700 ng/mL GH, and medium with hCF lentiGH had 120–140 ng/mL GH. No detectable hGH was found in coculture medium with cells infected with lentiV.

### 5.12 Gene Sequencing and Analysis

#### 5.12.1 Bulk RNA Sequencing

RNA sequencing was completed at the Cedars‐Sinai Genomics Core. Briefly, RNA samples were extracted from human intestinal organoids using RNeasy Micro kit (Qiagen), and a library was prepared with NEBNext Ultra II Directional RNA library prep kit (Illumina). Sequencing was performed on a NovaSeq 6000 (S10OD025052) at 25 M reads/sample using 50 × 50 paired‐end sequencing. Data quality was assessed using FastQC software. Sequences were aligned to the Genome Reference Consortium Human Build 38 (GRCh38) reference genome using STAR version 2.5.4b (Dobin et al. 2013). Genes with fewer than 10 counts were excluded from subsequent analyses. Differentially expressed genes were determined using DESeq2 version 1.36.0 (Love et al. 2014) using a ±2‐fold threshold and FDR adjusted *p* < 0.05.

#### 5.12.2 Library Preparation and Sequencing

Genomic DNA samples were assessed for concentration using a Qubit fluorometer (ThermoFisher Scientific) and for quality using the 4200 TapeStation (Agilent Technologies). 500 ng gDNA was used for library construction using the Illumina DNA Prep library preparation kit following the manufacturer's user guide. Five cycles of PCR were performed. Library concentration was measured via Qubit and library size via the TapeStation. Multiplexed libraries were pooled and sequenced on a NovaSeq 6000 (Illumina) at 25X coverage using 2 × 150 paired‐end sequencing.

#### 5.12.3 WGS Data Processing and Analysis

Using a whole‐genome sequencing approach, each sample was sequenced to approximately 30X genome coverage, then each group assessed SNPs, multinucleotide polymorphisms (MNP), small Indels, and larger insertions, deletions, translocations, and transversions (SV). SNPs, MNPs, and Indels were detected with the GATK best practices pipeline (v0.4.1.7), while larger SVs were detected with MANTA (v1.6.0). The genomic context and impact on coding regions were assessed using snpEff (v4.3). By cross‐referencing mutations exclusive to each experimental group with public databases such as the Human Genomes Project database, a list of candidate mutations was assembled whose presence/absence might impact overall mutational load or genome instability.

#### 5.12.4 Proteomics and Phosphoproteomics Analysis

Cells were lysed in ice‐cold lysis buffer (8 M urea, 25 mM Tris–HCl pH 8.6, 150 mM NaCl), containing 2 mM sodium beta‐glycerophosphate, 2 mM sodium fluoride, 2 mM sodium molybdate, 1 mM sodium orthovanadate, and Mini‐Complete EDTA‐free Protease Inhibitor Tablet (Roche Life Sciences) and then sonicated. Lysates were subjected to centrifugation (15,000 x g for 30 min at 4°C), supernatants transferred to a new tube, and protein concentration determined using a bicinchoninic acid assay (Pierce/ThermoFisher). DTT and iodoacetamide were added to reduce and alkylate, respectively. Protein samples were incubated overnight at 37°C with 1:100 (w/w) trypsin and trypsin digest was stopped the next day by the addition of 0.25% TFA (final v/v). Precipitated lipids were removed by centrifugation (3500 x g for 15 min), and peptides desalted over an Oasis HLB 60 mg plate (Waters). An aliquot containing ~20 μg of peptides was removed and labeled with Tandem‐Mass‐Tag (TMTpro) reagent (ThermoFisher). Once labeling efficiency was confirmed to be at least 95%, each reaction was quenched by the addition of hydroxylamine to a final concentration of 0.25% for 10 min, mixed, acidified with TFA to a pH of approximately 2, and desalted over an Oasis HLB 10 mg plate. The desalted multiplex was dried by vacuum centrifugation and separated by offline pentafluorophenyl‐based reversed‐phase HPLC fractionation as previously described (Grassetti et al. 2017). TMTpro‐labeled peptides were analyzed on an Orbitrap Lumos mass spectrometer (ThermoScientific) equipped with an Easy‐nLC 1200 system (ThermoScientific), and raw data were searched and processed (Papke et al. 2021). Peptide intensities were adjusted based on total TMT reporter ion intensity in each channel and log2 transformed. *p* values were calculated using a two‐tailed Student's *t*‐test (Tyanova et al. 2016). Phosphopeptide enrichment was achieved using a Fe‐NTA phosphopeptide enrichment kit (ThermoFisher) according to instructions provided by the manufacturer and desalted over an Oasis HLB 10 mg plate. Phosphopeptides were then labeled with TMTpro reagents, offline separated as described above, and analyzed on the Orbitrap Lumos. The probability of phosphorylation site localization was determined by PhosphoRS (Taus et al. 2011). Quantification and data analysis were carried out as above (Tyanova et al. 2016).

### 5.13 Bioinformatics Analysis

GO and pathway analysis were performed on proteins with phosphorylation sites that significantly increased or decreased in Webgestalt (Liao et al. 2019).

### 5.14 Statistical Analysis

Continuous data were tested across two groups with independent or paired Student's *t*‐test or ANOVA and included the factor of experimental replicate where appropriate (up to 4 replicates); in some experiments, mixed model regression was used to correct for random effects across experimental replicates, and post hoc testing was performed by Tukey test to control for multiple comparisons. Residuals were inspected to confirm fit of data, and where outliers were present, data were log‐transformed prior to analysis, and are presented as mean ± SEM. Differences were considered significant where *p* values were < 0.05. SAS v9.4 and GraphPad v9 software were used for analysis. Where data are graphed as percent of control (100%), statistical testing was performed on raw numbers.

### 5.15 Study Approval

Animal experiments were approved by the Cedars‐Sinai Institutional Animal Care and Use Committee (IACUC #005292). Generation of iPSC from fibroblasts obtained from healthy human volunteer donors was approved by the Cedars‐Sinai Medical Center Institutional Review Board (IRB #40182). Written informed consent was received from all donors prior to tissue collection.

## Author Contributions

V.C. and S.M. developed the hypothesis and wrote the manuscript. S.M. coordinated and directed the project. V.C., S.Z., T.A., and A.N.K. conducted experiments. R.A. analyzed RNA‐seq, WS, and phosphoproteomic data. A.N.K. and E.C.G. performed phosphoproteomic studies and analyzed the data. R.B. and C.W.V. generated human intestinal organoids. V.C., S.Z., and S.M. analyzed, discussed, and interpreted the data. All authors approved the manuscript before submission.

## Disclosure

The authors have nothing to report.

## Conflicts of Interest

The authors declare no conflicts of interest.

## Supporting information


**Figures S1–S9:** acel70187‐sup‐0001‐FiguresS1‐S9.pdf.

**Table S1:** acel70187‐sup‐0002‐TableS1.xlsx.

**Table S2:** acel70187‐sup‐0003‐TableS2.xlsx.

**Table S3:** acel70187‐sup‐0004‐TableS3.xlsx.

**Table S4:** acel70187‐sup‐0005‐TableS4.xlsx.

## Data Availability

Phospho‐omics/proteomics data that support the findings of this study are available in ProteomeXchange at proteomexchange.org, reference number PXD059561. Bulk RNA‐seq data are available in GEO, accession number GSE288661. WGS data are available in BioSample, accession numbers SAMN46795294, SAMN46795295, SAMN46795296, SAMN46795297, SAMN46795298, SAMN46795299.
