# Supplementary material for: Local Growth Hormone Facilitates Aging of the Colon Epithelial Microenvironment
Source: Aging Cell. 2025 Aug 5;24(10):e70187. doi: 10.1111/acel.70187 (PMC12507422; doi:10.1111/acel.70187)
Supplement: Supplementary file 1 — Figures S1–S9: acel70187‐sup‐0001‐FiguresS1‐S9.pdf. [file ACEL-24-e70187-s005.pdf]

Local growth hormone facilitates aging of the colon epithelial microenvironment

Vera Chesnokova, Svetlana Zonis, Richard Ainsworth, Tugce Apaydin, Christian Wong Valencia,

Elora C. Greiner, Robert Barrett, Arminja N. Kettenbach, Shlomo Melmed

Supplemental Information

Supplemental Figures Legends

SI Figure 1. npGH is induced in aging human colon tissue. Representative immunohistochemistry images of human colon specimens; scale bar = 200 µm. **(A)** npGH expression (brown) in specimens derived from a 23-year-old patient (top) and a 65-year-old patient (bottom). **(B)** Left, most cells expressing npGH (green) do not express Ki67 (pink). Right, multiple cells expressing npGH (green) also express p16 (pink). Arrows indicate areas of co-localization.

SI Figure 2. Autocrine GH triggers EMT and β-catenin nuclear translocation. (A-C) ImageJ quantification of protein expression normalized to loading controls from 3 independent experiments. **(A)** Cytoplasmic (CF) and nuclear (NF) fractions of hNCC infected with lentiV or lentiGH and analyzed 7 days after infection. **(B,C)** EMT markers in hNCC line #1 and line #2 infected with lentiV or lentiGH. Results are depicted as mean ± SEM of triplicate measurements. *p<0.05, **p<0.01. Microscope images depicting **(D)** Migration and **(E)** invasion of hNCC infected with lentiV or lentiGH. Scale bar=100 µm.

SI Figure 3. GH triggers cytoskeleton rearrangement consistent with migration in hNCC infected with lentiGH. 200× magnification images depict the appearance of long cellular extension (arrows) in cells.

SI Figure 4. Paracrine GH triggers β-catenin nuclear translocation and EMT. (A-B) ImageJ quantification of protein expression normalized to loading controls from 3 independent experiments. **(A)** Cytoplasmic (CF) and nuclear (NF) fractions of hNCC co-cultured with hNCC infected with lentiV or lentiGH and analyzed 7 days later. **(B)** EMT markers in hNCC co-cultured with hNCC infected with lentiV or lentiGH and analyzed 7 days after infection. **(C)**

Western blot and **(D)** ImageJ quantification of protein expression in hNCC line #2 co-cultured with hNCC infected with lentiV or lentiGH and analyzed 7 days after infection. In A,B,D results are depicted as mean ± SEM of triplicate measurements. *$p<0.05$, **$p<0.01$. **(E-F)** Microscope images depicting **(E)** migration and **(F)** invasion of hNCC infected with lentiV or lentiGH. Scale bar=100 µm.

SI Figure 5. Microscope images depicting **(A)** migration and **(B)** invasion of hNCC infected with lentiV or lentiGH. Scale bar=100 µm.

SI Figure6. Paracrine GH triggers EMT in human intestinal organoids and murine colon issue. ImageJ quantification of protein expression normalized to loading controls from 3 independent experiments. **(A)** EMT markers in organoids co-cultured for 1 month with organoids expressing lentiV or lentiGH. **(B)** EMT markers in the colon tissue of mice carrying GH-secreting or vector-secreting xenografts. Results are depicted as mean ± SEM. *$p<0.05$, **$p<0.01$.

SI Figure 7. EMT markers in the colon of WT and GHRKO mice. (A) Western blot. **(B)** ImageJ quantification of protein expression normalized to loading controls. Results are depicted as mean ± SEM.

SI Figure 8. RNA-seq results. (A) GO and **(B)** KEGG analysis of RNA-seq results show paracrine GH significantly affected several ECM-associated and focal adhesion pathways.

SI Figure 9. Paracrine GH alters expression of cytoskeleton proteins and myosins. ImageJ quantification of protein expression normalized to loading controls from 3 independent experiments. **(A)** Cytoskeleton proteins in organoid cells cultured in the presence of lentiV or

lentiGH. **(B)** Cytoskeleton proteins in the colon tissue of WT and GHRKO mice. **(C)** Myosin expression in hNCC treated with 500 ng/ml GH for 6 hours. Results are depicted as mean ± SEM of triplicate measurements. *p<0.05, **p<0.01.

Supplemental Tables Legends

SI Table1. Comparative Analysis of gene expression in organoids exposed to paracrine GH vs. vector. The table includes adjusted p-value and log2 (fold-change). In pathway enrichment analysis (Reactome), pathways depicted in SI Fig.8 are highlighted in yellow. Pathways are ranked based on statistical significance and biological impact.

SI Table 2. Quantitative changes in the proteome and phosphoproteome in hNCC treated with GH for 6 and 24 hours. Experiments were performed in triplicate and results are presented as raw values, log2 ratio (fold-change) between untreated control vs. treated cells.

SI Table 3. Pathway enrichment analysis of phosphoproteome in hNCC treated with GH for 24 hours. Pathways depicted in Fig.5B are highlighted in yellow. Pathways are ranked based on statistical significance and biological impact.

SI Table 4. Quantitative changes in the myosin proteins in hNCC treated with GH for 6 hours. Experiment was performed in triplicate and results are presented as raw values, log2 ratio between untreated control vs. treated cells.

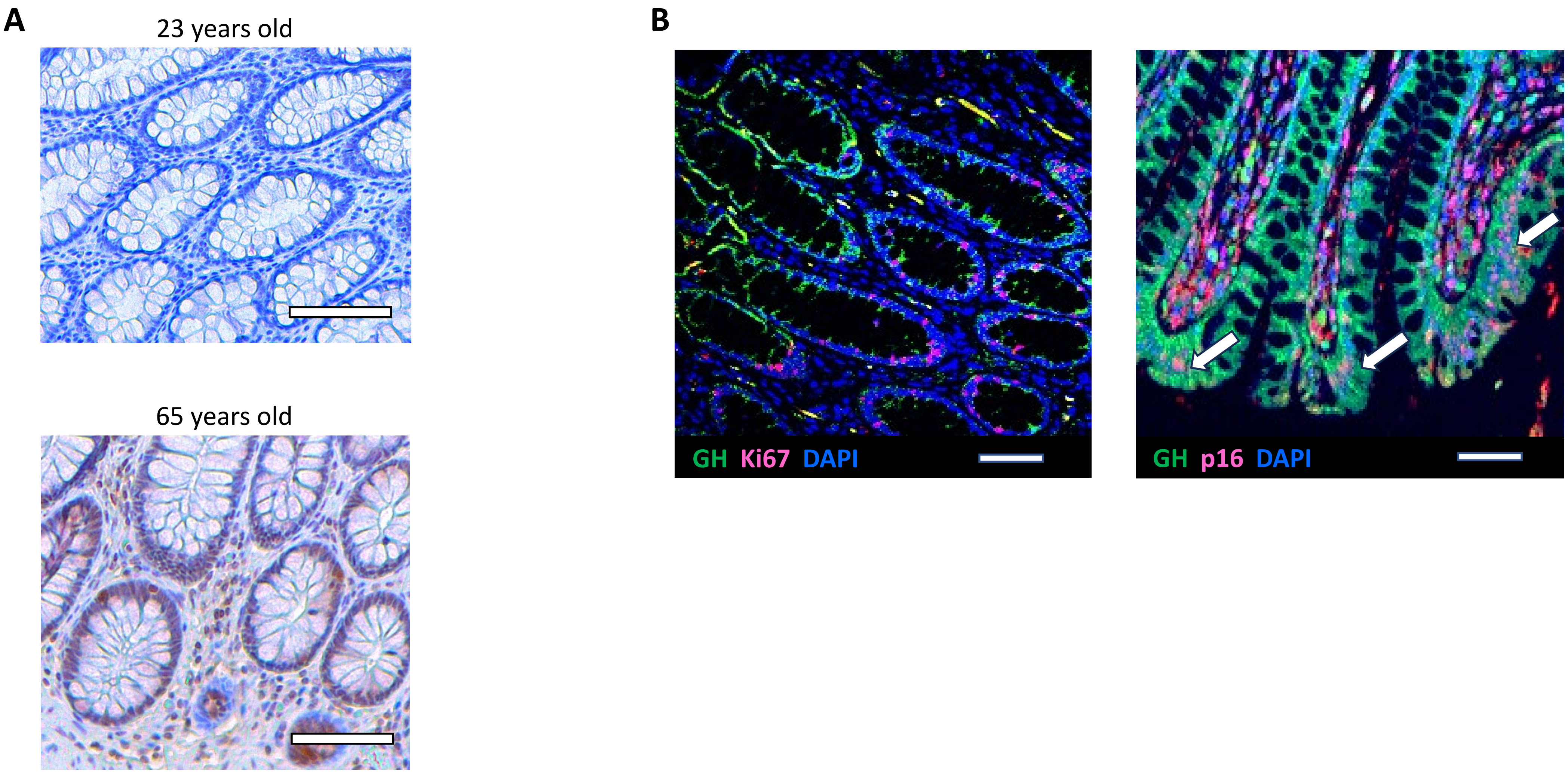

SI Figure 1

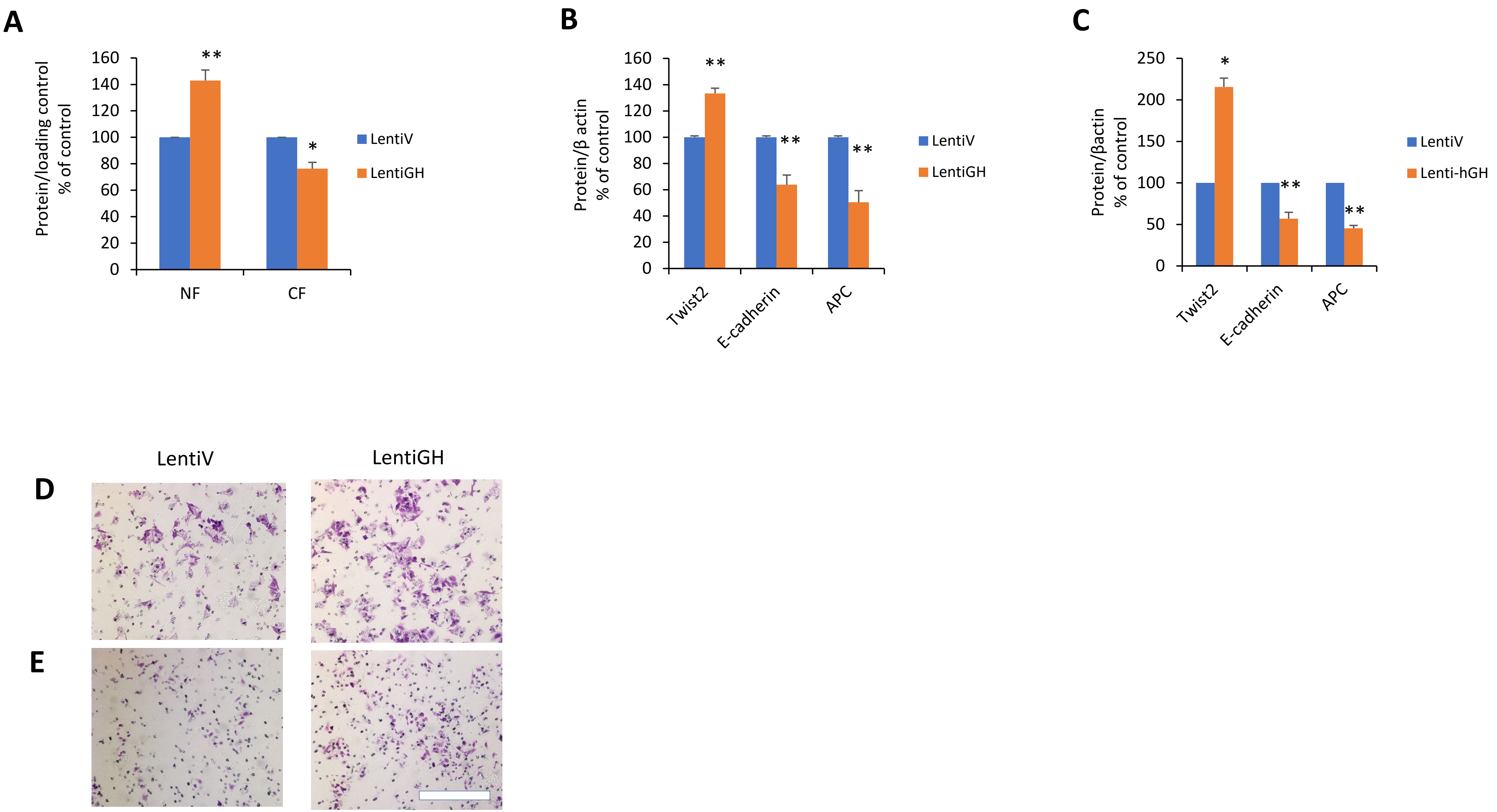

A
Protein/loading control % of control
160
140
120
100
80
60
40
20
0
NF
CF
**
*
LentiV
LentiGH
B
Protein/β actin % of control
Twist2
E-cadherin
APC
LentiV
LentiGH
C
Protein/βactin % of control
250
200
150
100
50
0
Twist2
E-cadherin
APC
LentiV
Lenti-hGH
LentiV
LentiGH
D
E

SI Figure 2

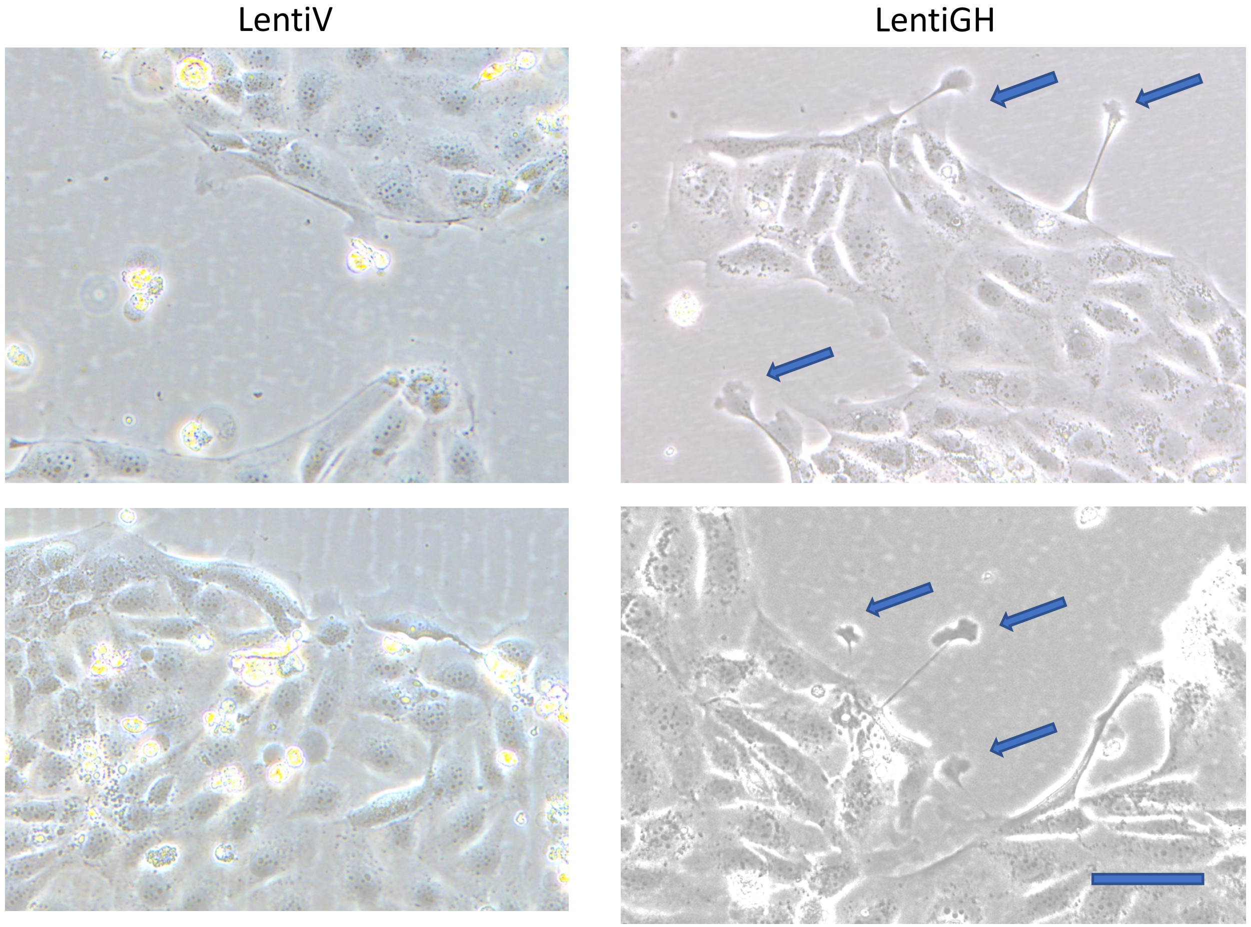

SI Figure 3

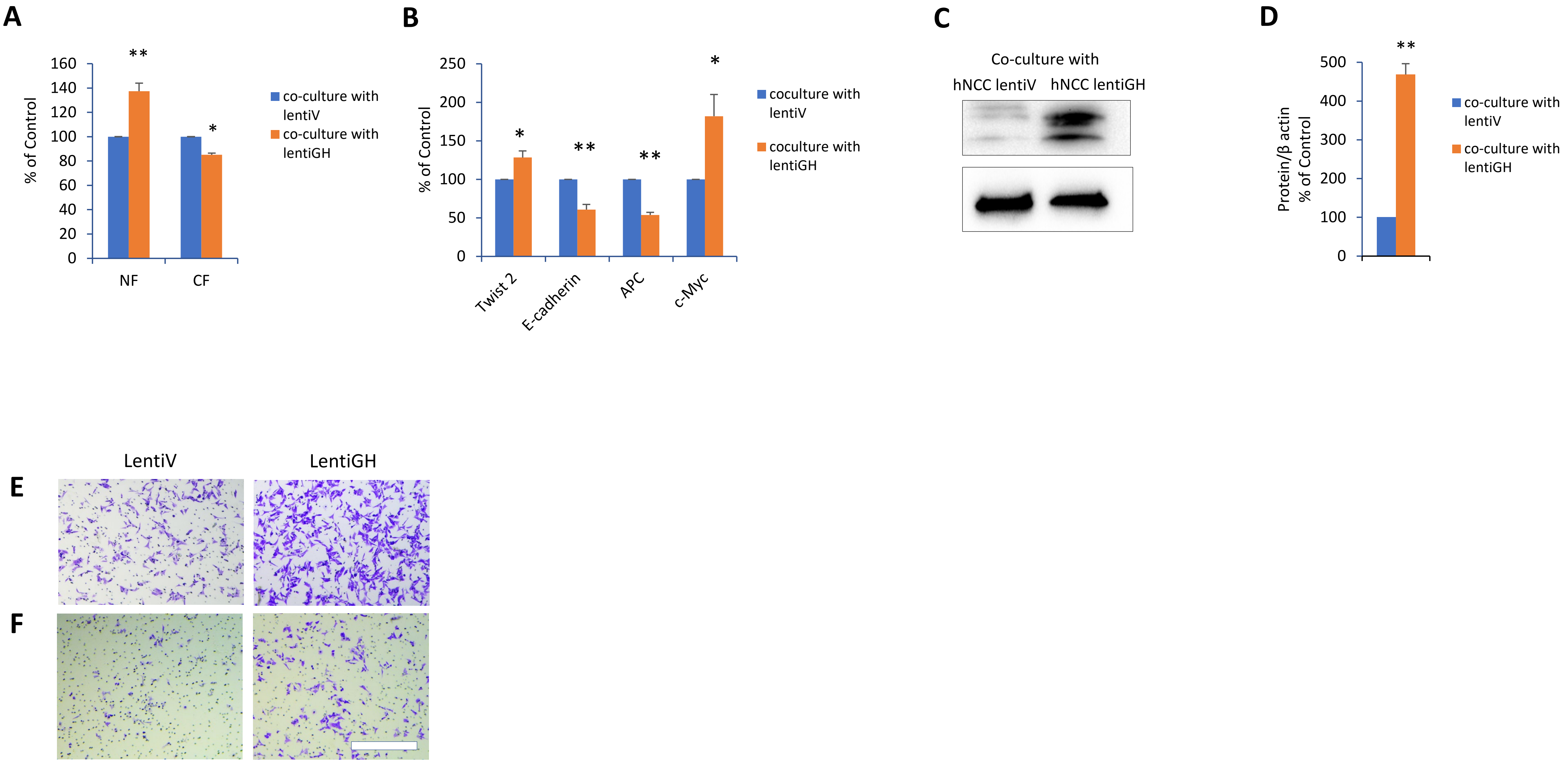
A
160
140
120
100
80
60
40
20
0
% of Control
**
*
NF
CF
co-culture with lentiV
co-culture with lentiGH
B
250
200
150
100
50
0
% of Control
*
**
**
*
Twist 2
E-cadherin
APC
c-Myc
coculture with lentiV
coculture with lentiGH
C
Co-culture with
hNCC lentiV
hNCC lentiGH
D
500
400
300
200
100
0
Protein/β actin
% of Control
**
co-culture with lentiV
co-culture with lentiGH
LentiV
LentiGH
E
F

SI Figure 4

Co-culture with

hCF lentiV hCF lentiGH

A

B

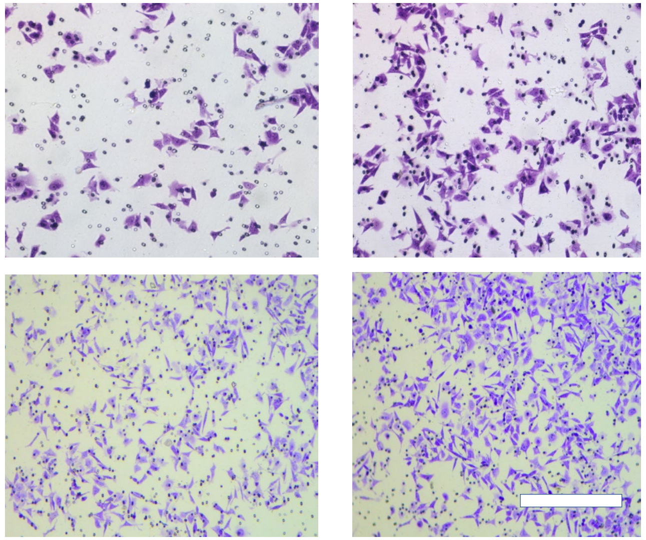

SI Figure 5

A

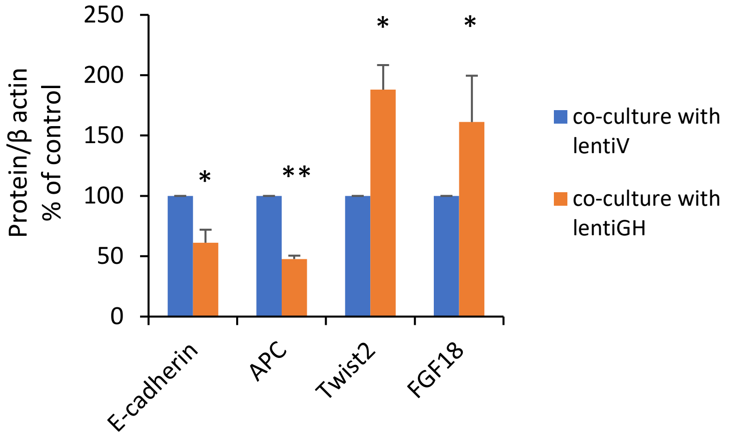

B

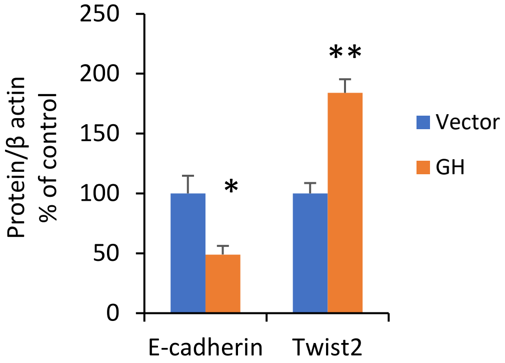

SI Figure 6

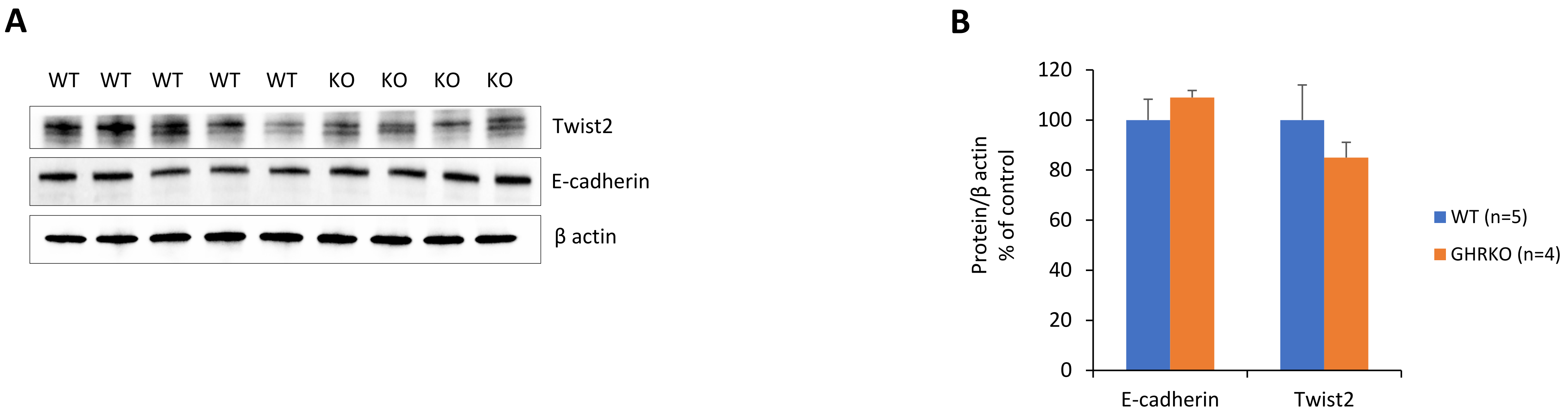
A
WT WT WT WT WT KO KO KO KO
Twist2
E-cadherin
β actin
B
Protein/β actin
% of control
120
100
80
60
40
20
0
E-cadherin
Twist2
WT (n=5)
GHRKO (n=4)

SI Figure 7

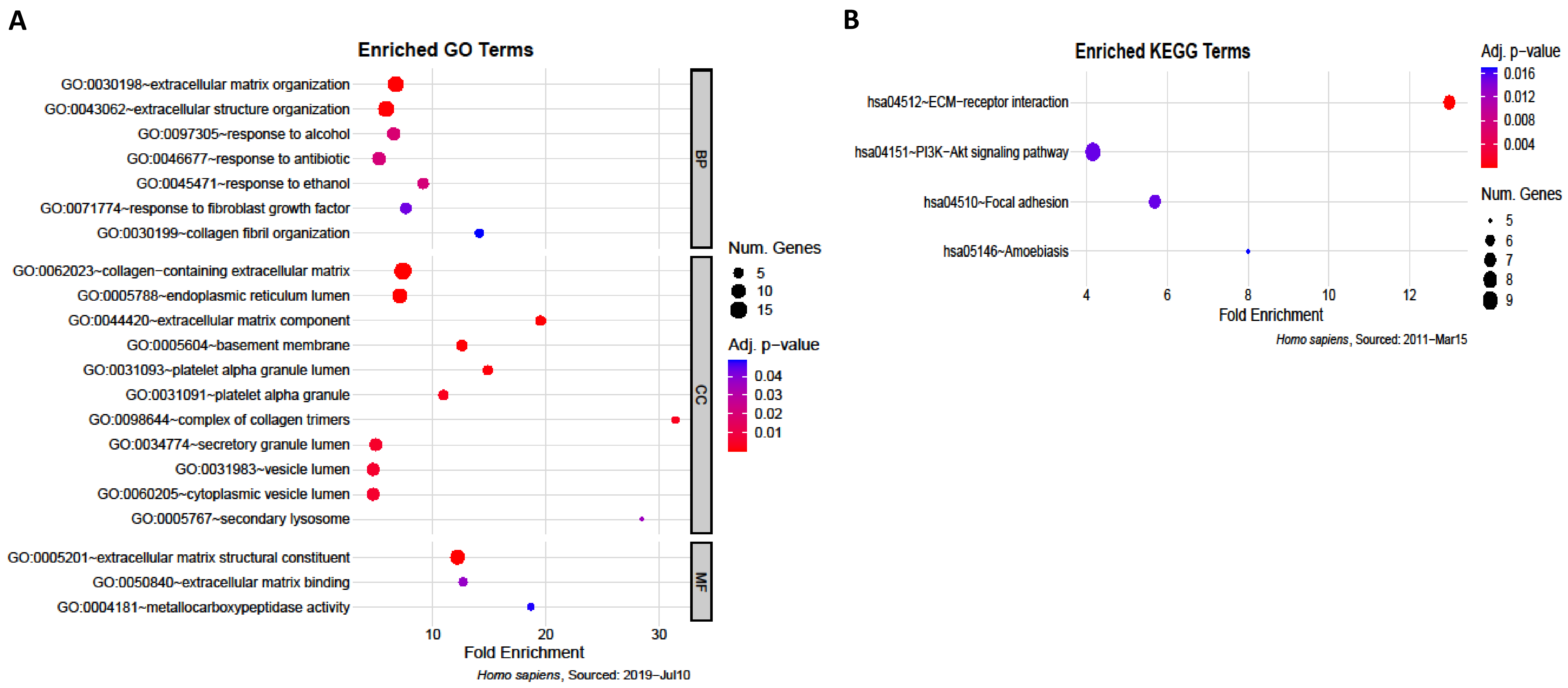
A
Enriched GO Terms
GO:0030198~extracellular matrix organization
GO:0043062~extracellular structure organization
GO:0097305~response to alcohol
GO:0046677~response to antibiotic
GO:0045471~response to ethanol
GO:0071774~response to fibroblast growth factor
GO:0030199~collagen fibril organization
GO:0062023~collagen−containing extracellular matrix
GO:0005788~endoplasmic reticulum lumen
GO:0044420~extracellular matrix component
GO:0005604~basement membrane
GO:0031093~platelet alpha granule lumen
GO:0031091~platelet alpha granule
GO:0098644~complex of collagen trimers
GO:0034774~secretory granule lumen
GO:0031983~vesicle lumen
GO:0060205~cytoplasmic vesicle lumen
GO:0005767~secondary lysosome
GO:0005201~extracellular matrix structural constituent
GO:0050840~extracellular matrix binding
GO:0004181~metallocarboxypeptidase activity
BP
CC
MF
10
20
30
Fold Enrichment
Homo sapiens, Sourced: 2019−Jul10
Num. Genes
5
10
15
Adj. p−value
0.04
0.03
0.02
0.01
B
Enriched KEGG Terms
hsa04512~ECM−receptor interaction
hsa04151~PI3K−Akt signaling pathway
hsa04510~Focal adhesion
hsa05146~Amoebiasis
4
6
8
10
12
Fold Enrichment
Homo sapiens, Sourced: 2011−Mar15
Adj. p−value
0.016
0.012
0.008
0.004
Num. Genes
5
6
7
8
9

SI Figure 8

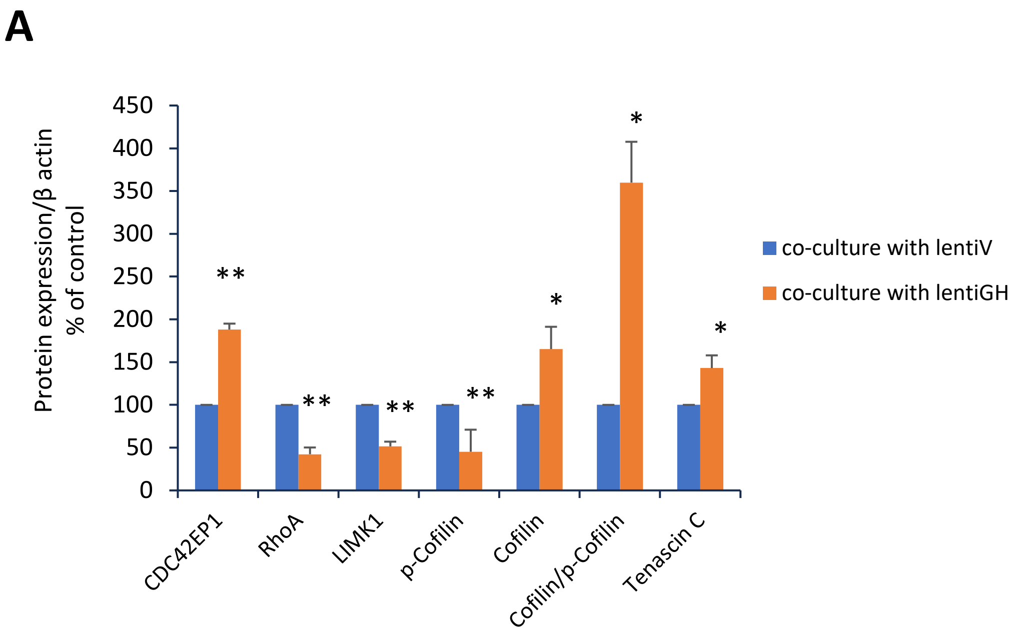

A
Protein expression/β actin
% of control
0
50
100
150
200
250
300
350
400
450
**
**
**
**
*
*
*
CDC42EP1
RhoA
LIMK1
p-Cofilin
Cofilin
Cofilin/p-Cofilin
Tenascin C
co-culture with lentiV
co-culture with lentiGH

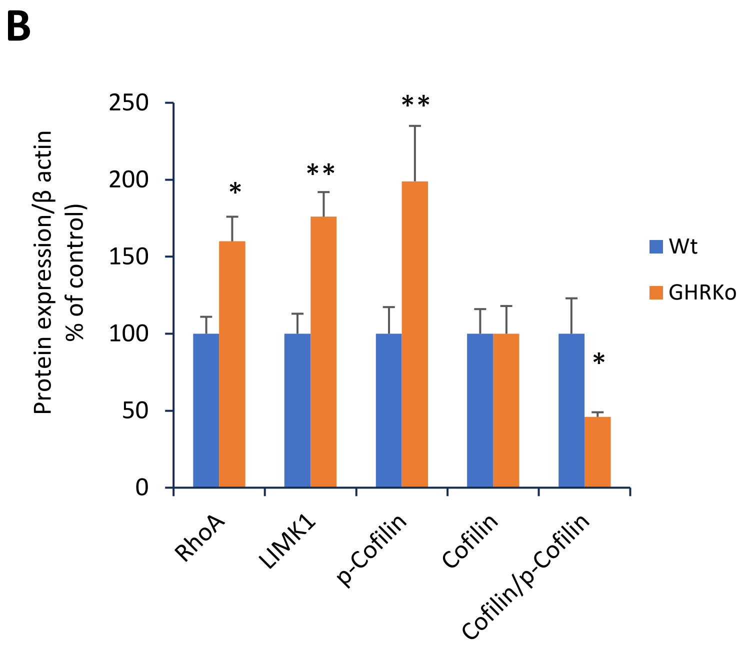

B
Protein expression/β actin
% of control)
0
50
100
150
200
250
*
**
**
*
RhoA
LIMK1
p-Cofilin
Cofilin
Cofilin/p-Cofilin
Wt
GHRKo

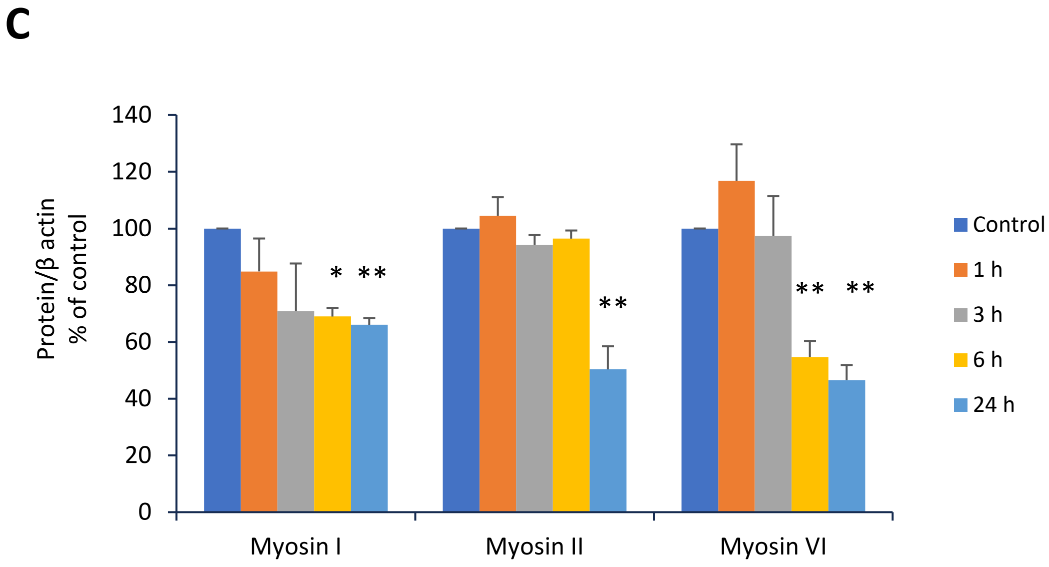

C
Protein/β actin
% of control
0
20
40
60
80
100
120
140
*
**
**
**
**
Myosin I
Myosin II
Myosin VI
Control
1 h
3 h
6 h
24 h

SI Figure 9
